# The Balance between Actin-Bundling Factors Controls Actin Architecture in Pollen Tubes

**DOI:** 10.1016/j.isci.2019.05.026

**Published:** 2019-05-25

**Authors:** Ruihui Zhang, Xiaolu Qu, Meng Zhang, Yuxiang Jiang, Anbang Dai, Wanying Zhao, Dai Cao, Yaxian Lan, Rong Yu, Hongwei Wang, Shanjin Huang

**Affiliations:** 1 Center for Plant Biology, School of Life Sciences, Tsinghua University, Beijing 100084, China; 2 Ministry of Education Key Laboratory of Protein Sciences, Tsinghua-Peking Joint Center for Life Sciences, Beijing Advanced Innovation Center for Structural Biology, School of Life Sciences, Tsinghua University, Beijing 100084, China; 3 School of Life Sciences, Capital Normal University, Beijing 100048, China

**Keywords:** Biological Sciences, Cell Biology, Functional Aspects of Cell Biology, Plant Biology

## Abstract

How actin-bundling factors cooperatively regulate shank-localized actin bundles remains largely unexplored. Here we demonstrate that FIM5 and PLIM2a/PLIM2b decorate shank-localized actin bundles and that loss of function of *PLIM2a* and/or *PLIM2b* suppresses phenotypes associated with *fim5* mutants. Specifically, knockout of *PLIM2a* and/or *PLIM2b* partially suppresses the disorganized actin bundle and intracellular trafficking phenotype in *fim5* pollen tubes. PLIM2a/PLIM2b generates thick but loosely packed actin bundles, whereas FIM5 generates thin but tight actin bundles that tend to be cross-linked into networks *in vitro*. Furthermore, PLIM2a/PLIM2b and FIM5 compete for binding to actin filaments *in vitro*, and PLIM2a/PLIM2b decorate disorganized actin bundles in *fim5* pollen tubes. These data together suggest that the disorganized actin bundles in *fim5* mutants are at least partially due to gain of function of *PLIM2a*/*PLIM2b*. Our data suggest that the balance between FIM5 and PLIM2a/PLIM2b is crucial for the normal bundling and organization of shank-localized actin bundles in pollen tubes.

## Highlights

• The transcription of *PLIM2a* and *PLIM2b* is upregulated in *fim5* pollen tubes
• Downregulation of *PLIM2a* and/or *PLIM2b* suppresses the defects in *fim5* pollen tubes
• Both FIM5 and PLIM2a/PLIM2b decorate shank-localized actin filaments
• FIM5 can inhibit the binding of PLIM2a and PLIM2b to actin filaments

## Graphical Abstract

**Figure:**
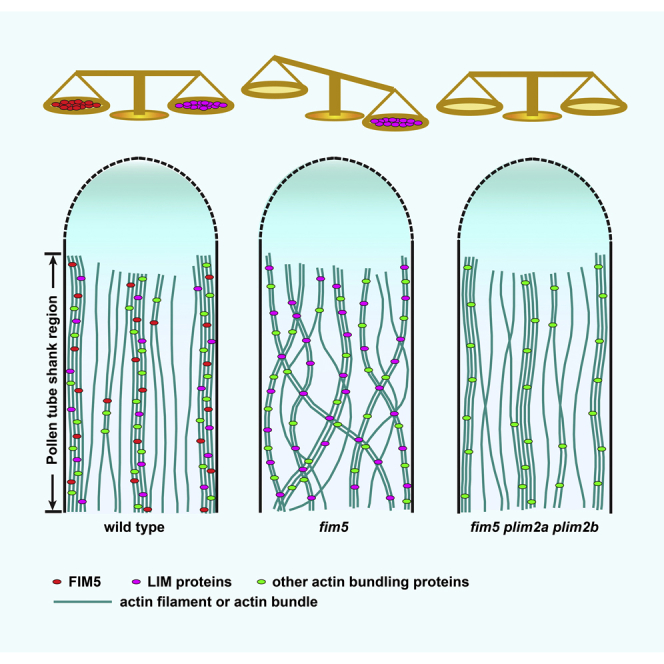

## Introduction

The actin cytoskeleton has been implicated in numerous fundamental physiological cellular processes, such as cell motility, cell division, cytokinesis, cell expansion, and intracellular trafficking (Pollard, 2016, Pollard and Cooper, 2009). Within cells, actin filaments are normally packed into higher-order structures, such as tight actin bundles and loose actin networks, which perform distinct cellular functions. Well-organized actin structures have been revealed in pollen tubes. Specifically, within the apical and subapical regions, actin filaments are highly dynamic and are directly involved in the regulation of pollen tube growth and turning (Cheung et al., 2010, Qu et al., 2017). Within the shank region of angiosperm pollen tubes, actin filaments form parallel actin bundles with their barbed ends facing tipward at the cortex and backward within the middle region (Lenartowska and Michalska, 2008). This unique organization pattern of longitudinal actin bundles together with the barbed-end-directed myosin XIs (Madison et al., 2015) generates the reverse fountain pattern of cytoplasmic streaming and drives various intracellular trafficking events in pollen tubes (Chebli et al., 2013, Chen et al., 2009, Cheung and Wu, 2008, Fu, 2015, Guan et al., 2013, Qin and Yang, 2011, Qu et al., 2015, Ren and Xiang, 2007, Staiger et al., 2010, Vidali and Hepler, 2001). However, the molecular mechanism by which the shank-oriented actin bundles are generated and maintained in pollen tubes remains incompletely understood.

Within cells, the generation and maintenance of certain higher-order actin structures are coordinately regulated by numerous actin-binding proteins, such as actin-nucleation factors, actin-severing proteins, and actin-bundling factors (Davidson and Wood, 2016, Kovar et al., 2011, Michelot and Drubin, 2011). Among them, actin-bundling factors dictate the formation of specific higher-order actin structures, including tight actin bundles (Huang et al., 2015, Thomas et al., 2009). Several types of actin-bundling factors with distinct biochemical activities and regulatory functions may coexist within the cytoplasm of certain cells. It remains largely unexplored how these different actin-bundling factors coordinately regulate the formation and maintenance of actin bundles with specific features. Several actin-bundling factors, including fimbrin, villin, and LIM domain-containing proteins (Gui et al., 2014, Nakayasu et al., 1998, Qu et al., 2013, Su et al., 2012, Su et al., 2017, Thomas et al., 2007, Wang et al., 2008, Wu et al., 2010, Yokota and Shimmen, 1999, Yokota et al., 2003), have been implicated in the generation of longitudinal actin bundles within the shank region of pollen tubes. However, almost nothing is known about how they might coordinately regulate the construction and maintenance of shank-oriented actin bundles. Previous studies showed that *Arabidopsis* FIMBRIN5 (FIM5) regulates the construction of shank-localized actin bundles and the apical actin structure in pollen tubes (Su et al., 2012, Wu et al., 2010, Zhang et al., 2016a, Zhang et al., 2016b). Surprisingly, it was shown that *fim5* pollen tubes are filled with uniform intermediate-sized but disorganized actin bundles (Su et al., 2012, Wu et al., 2010, Zhang et al., 2016a, Zhang et al., 2016b). It is quite perplexing that loss of function of a *bona fide* actin bundler causes such an actin bundle phenotype rather than the simple reduction in the extent of actin filament bundling as expected in pollen tubes. It is possible that other biochemically distinct actin-bundling factors take over the FIM5-binding sites on actin filaments, and this leads to the formation of uniform intermediate-sized but disorganized actin bundles in *fim5* pollen tubes.

We speculated that if the actin bundle phenotype of *fim5* pollen tubes is caused by the substitution of other biochemically distinct actin-bundling factors, the transcription of the genes encoding those factors might be altered in *fim5* pollen tubes due to some unknown feedback regulatory mechanism. Therefore we initially examined the level of transcripts of other actin-bundling factors in *fim5* pollen and found that *PLIM2a* and *PLIM2b* transcripts were upregulated significantly in *fim5* pollen when compared with wild-type (WT). We found that loss of function of *PLIM2a* and/or *PLIM2b* partially suppresses the shank-localized actin bundle and pollen tube growth phenotypes in *fim5* pollen tubes. *In vitro* biochemical data showed that PLIM2a and PLIM2b generate loosely packed but thicker actin bundles when compared with the thin and tight actin bundles generated by FIM5, and FIM5 competes with PLIM2a or PLIM2b for binding to actin filaments. Our results suggest that maintaining the balance between these two types of actin-bundling factor is necessary for the generation of properly organized actin bundles in the shank region of pollen tubes. Our study thus substantially enhances our understanding of the molecular mechanism underlying the generation and maintenance of longitudinal actin bundles in the shank region of pollen tubes.

## Results

### The Expression of *PLIMs* Is Upregulated in *fim5* Mutants and Loss of Function of *PLIM2a* and/or *PLIM2b* Suppresses the Phenotype Associated with *fim5*

**Figure 1:**
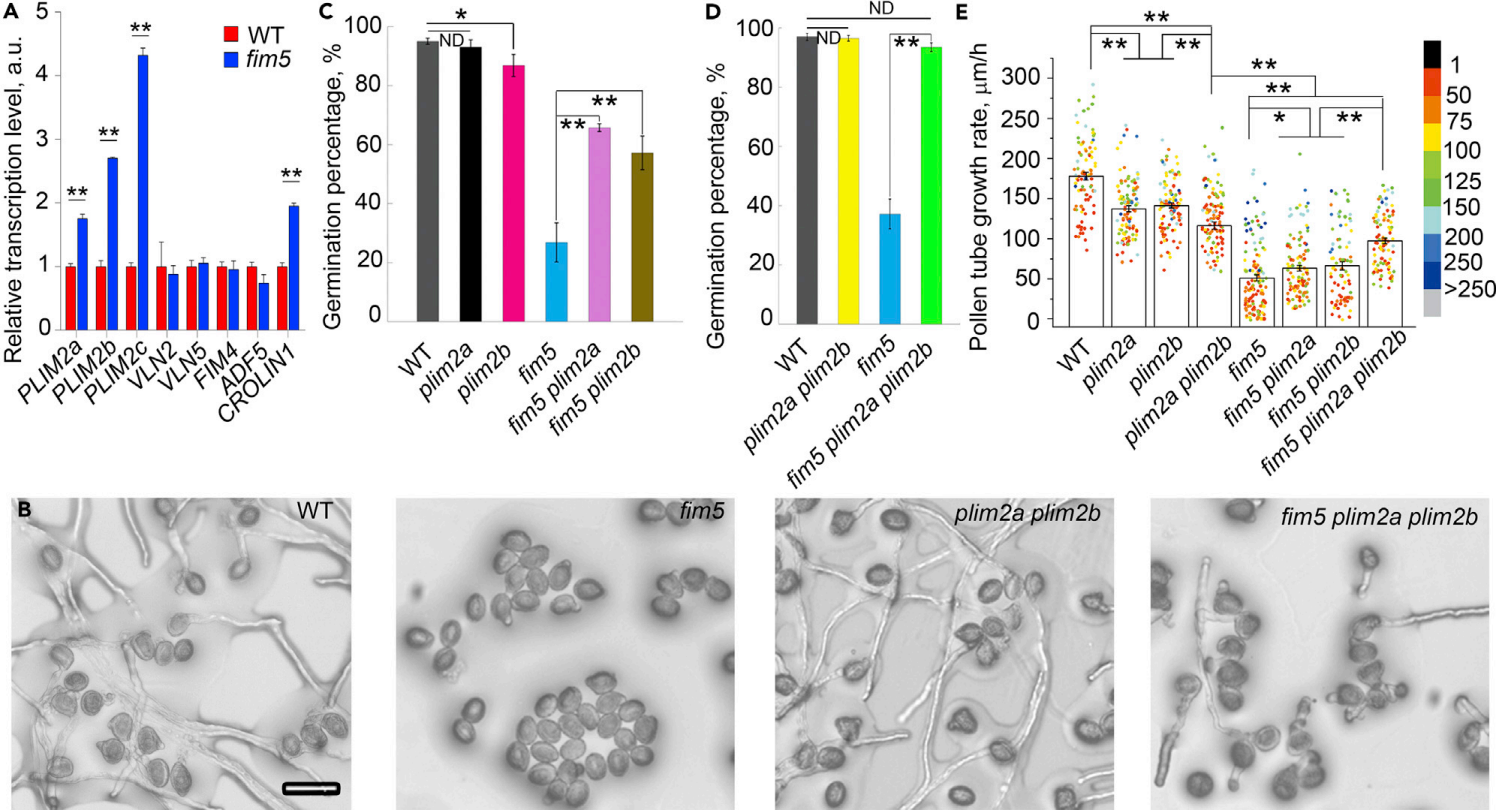
The Expression of *PLIMs* Is Upregulated in *fim5*, and Loss of Function of *PLIM2a* and/or *PLIM2b* Suppresses the Phenotypes in *fim5* (A) qRT-PCR analysis to determine the relative amount of transcripts of several actin-bundling factors encoding genes in pollen derived from WT and *fim5* plants. The relative transcript levels are presented as mean ± SD; *n* = 3, **p < 0.01 by Student’s *t*-test. (B) Micrographs of pollen after germination for 3 h *in vitro*. Scale bar, 50 μm. (C) Loss of function of *PLIM2a* or *PLIM2b* enhances pollen germination in *fim5*. Error bar represents SE. *p < 0.05, **p < 0.01 by χ^2^ test. ND, no significant difference. *n* = 3. At least 500 pollen grains were counted for each genotype. (D) Loss of function of *PLIM2a* and *PLIM2b* enhances pollen germination in *fim5*. Error bar represents SE. **p < 0.01 by χ^2^ test. ND, no significant difference. *n* = 3. At least 500 pollen grains were counted for each genotype. (E) Loss of function of *PLIM2a* and/or *PLIM2b* enhances pollen tube growth in *fim5*. Error bar represents SE. *p < 0.05, **p < 0.01 by Student’s *t*-test. ND, no significant difference. At least three independent experiments were performed and one typical result was shown. At least 80 pollen tubes were counted for each genotype.

We initially examined the transcript levels of several actin-bundling factors in *fim5* pollen and compared them with the levels in WT pollen. Interestingly, we found that the transcript levels of *PLIM2a*, *PLIM2b,* and *PLIM2c* are upregulated in *fim5* pollen (Figure 1A). Considering that the transcription of *CROLIN1* (Jia et al., 2013) is also upregulated in *fim5* pollen (Figure 1A), the upregulation in the transcription of *PLIMs* in *fim5* pollen might not be completely specific. However, the upregulation of the transcription of all three *PLIMs* inspired us to speculate that the actin-based phenotype in *fim5* might to some extent be due to the upregulation of *PLIMs*. It was reported that PLIM2c is biochemically distinct from PLIM2a and PLIM2b as it is the only *Arabidopsis* LIM to clearly respond to Ca^2+^ (Papuga et al., 2010). As we are interested in understanding the role of FIM5 in regulating actin bundles in the shank where there is no obvious fluctuation of cytosolic [Ca^2+^] during normal pollen tube growth (Diao et al., 2018, Messerli et al., 2000, Pierson et al., 1994), we focused on characterizing the functional coordination of PLIM2a/PLIM2b with FIM5 in the regulation of shank-localized actin bundles. We then hypothesized that loss of function of *PLIM2a* and *PLIM2b* may suppress the phenotype in *fim5*. We therefore analyzed the transfer DNA insertion mutants of *PLIM2a* and *PLIM2b* and found that they redundantly regulate pollen tube growth (Figure S1). We then generated *fim5 plim2a* and *fim5 plim2b* double mutants as well as *fim5 plim2a plim2b* triple mutants. We found that the pollen germination percentage and tube growth rates were increased in pollen derived from *fim5 plim2a*, *fim5 plim2b,* and *fim5 plim2a plim2b* mutant plants when compared with that derived from *fim5* plants (Figures 1B–1E). This suggests that loss of function of *PLIM2a* and/or *PLIM2b* suppresses the pollen germination and pollen tube growth phenotypes associated with *fim5*. However, we found that loss of function of *PLIM2a* and/or *PLIM2b* does not suppress the pollen tube width phenotype in *fim5* pollen tubes (Figure S2). Together these results indicate that the phenotype associated with *fim5* is at least partially due to the gain of function of *PLIM2a* and *PLIM2b* in pollen tubes.

### Loss of Function of *PLIM2a* and *PLIM2b* Suppresses Actin Bundle Phenotypes in the Shank Region of *fim5* Pollen Tubes

**Figure 2:**
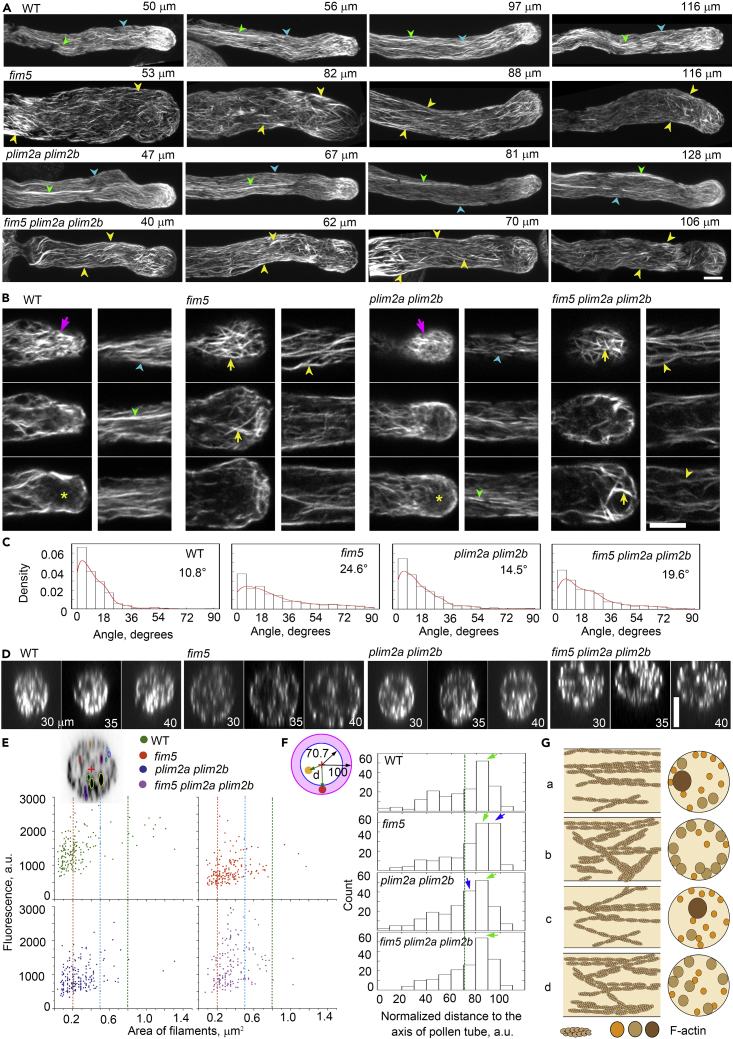
The Actin Bundle Phenotype is Alleviated within the Shank Region of *fim5* Pollen Tubes When the Expression of *PLIM2a* and/or *PLIM2b* is Abolished (A) Actin filaments in pollen tubes derived from WT, *fim5*, *plim2a plim2b,* and *fim5 plim2a plim2b* mutants of different lengths. Heavy actin bundle and fine actin structures are indicated by green and blue arrowheads in WT and *plim2a plim2b* pollen tubes, respectively. Uniformly sized but disorganized actin bundles in *fim5* and *fim5 plim2a plim2b* pollen tubes are indicated by yellow arrowheads. (B) Images showing actin filaments within the apical region (10 μm from the tip) and shank region (30–40 μm from the tip). Apical actin filaments (left columns) are indicated by magenta arrows in WT and *plim2a plim2b* pollen tubes and yellow arrows in *fim5* and *fim5 plim2a plim2b* pollen tubes. Actin filaments are arranged into bright actin structures within the apical region of WT and *plim2a plim2b* pollen tubes, whereas they are arranged into uniformly sized but disorganized actin bundles with a moderate extent of bundling within the apical region of *fim5* and *fim5 plim2a plim2b* pollen tubes. The middle regions with less abundant actin filaments are indicated by yellow asterisks within the apical region of WT and *plim2a plim2b* pollen tubes. Within the shank region (right columns), thick and thin actin bundles of WT and *plim2a plim2b* pollen tubes are indicated with green and blue arrowheads, respectively, whereas the intermediate-sized actin bundles in *fim5* and *fim5 plim2a plim2b* pollen tubes are indicated by yellow arrowheads. It suggests that the alignment of actin filaments is slightly recovered in *fim5 plim2a plim2b* pollen tubes compared with that in *fim5* pollen tubes. (C) Histograms of the angles formed between the shank-localized actin bundles and the growth axis of pollen tubes. The values of average angles of each genotype are indicated in the image. At least three independent experiments were performed and one typical result was shown. More than 700 actin bundles were measured for each genotype. (D) Transverse sections of pollen tubes within the shank region. The distances of the transverse sections from the pollen tube tip are indicated in images. (E) Quantification of the area and fluorescence intensity of actin structures within transverse sections from the shank region of pollen tubes. The inset image shows the method of measuring the area and fluorescence intensity of actin structures (different colored circles) and their distances to the center (indicated by the red plus sign). The fluorescence intensity was plotted versus the area of actin structures. Red, blue, and green dashed lines indicate actin filament areas of 0.2, 0.5, and 0.8 μm^2^, respectively. At least three independent experiments were performed and one typical result was shown. More than 170 actin bundles were measured for each genotype. (F) Histogram of the distances between actin structures and the center of cross sections derived from the shank region of pollen tubes. The method of distance measurement was described in (E). As the size of pollen tubes varies, the measured distances were normalized to the radius of pollen tubes before generating the plot. The image (left panel) is the schematic diagram showing that the area of the inner circle (white colored) is equal to that in the outside annulus (purple colored). The radius of the inner circle is 70.7, whereas the radius of the transverse section is normalized to 100. Yellow and red colored dots indicate actin structures, and the value of “d” indicates the distance of actin structures to the center. The histogram was generated via plotting the count versus the values of “d” of actin structures. The green dashed line indicates the radius at 70.7. Green arrows indicate the peaks in the histogram, and blue arrows indicate the fluorescence intensity of actin structures in mutant pollen tubes that are obviously different from that in WT pollen tubes. In terms of the role of actin in driving cytoplasmic streaming in angiosperm pollen tubes, actin structures within the outside annule (corresponding to cortical actin structures in longitudinal sections) and actin structures in inner circle (corresponding to middle actin structures in longitudinal sections) are more relevant to the tipward and backward movement of vesicles, respectively. At least three independent experiments were performed and one typical result was shown. More than 170 actin bundles were measured for each genotype. (G) Schematic describing the distribution of actin structures in the shank region of WT (a), *fim5* (b), *plim2a plim2b* (c), and *fim5 plim2a plim2b* (d) pollen tubes. Left and right panels are longitudinal and transverse sections of pollen tubes, respectively. In the shank region of WT pollen tubes, both thin and thick actin exist, and they are aligned longitudinally. The shank region of *fim5* pollen tubes is filled with uniformly sized but disorganized actin bundles, and they are comparatively more concentrated at the cortex. Compared with *fim5* pollen tubes, *fim5 plim2a plim2b* pollen tubes have fewer actin bundles at the cortex and they are comparatively straight. Scale bars, 5 μm in all images.

Next, we sought to examine how the loss of function of *PLIM2a* and/or *PLIM2b* affects the organization of the actin cytoskeleton in *fim5* pollen tubes. Previous observation showed that *fim5* pollen tubes are much wider at the early growing stage, but the width is almost normal at the late growing stage (Su et al., 2012). We visualized actin filaments within pollen tubes of different lengths. Indeed, the morphology of *fim5* pollen tubes is irregular, as the base of pollen tube is much wider (Figure S3A). Short *fim5* pollen tubes also exhibit a more severe disorganized actin bundle phenotype (Figure S3B). To reveal the effect of loss of function of *PLIM2a* and/or *PLIM2b* on the actin cytoskeleton in *fim5* pollen tubes, we selected pollen tubes shorter than 150 μm for detailed analysis and comparison. We found that in WT pollen tubes of different lengths, actin filaments form two kinds of actin bundle structures—heavy and fine—that align longitudinally within the shank (Figure 2A). Similar to previous findings, we found that *fim5* pollen tubes are filled with uniform intermediate-sized actin bundles that are disorganized in terms of their orientation relative to the growth axis of pollen tubes (Figure 2A). Although PLIM2a and PLIM2b are bona fide actin bundlers (see Papuga et al., 2010), the overall organization of actin bundles in the shank region of *plim2a plim2b* pollen tubes was very similar to that in WT pollen tubes and only showed slight disorganization in terms of longitudinal orientation (Figure 2A). Interestingly, we found that the actin bundle structures in the shank region of *fim5 plim2a plim2b* pollen tubes were arranged more regularly compared with those in *fim5* pollen tubes (Figure 2A). Although FIM5 and PLIM2a/PLIM2b are distributed along the entire pollen tube (see below), we found that there is no dramatic difference in the organization of apical actin filaments between *fim5* and *fim5 plim2a plim2b* pollen tubes (Figure 2B). This suggests that FIM5 and PLIM2a/PLIM2b differentially coordinate within different regions of the pollen tube.

The irregular arrangement of actin bundles within the shank region of pollen tubes was assessed by measuring the angles formed between the bundles and the pollen tube growth axis. The angles were increased in *fim5* and *plim2a plim2b* pollen tubes (Figure 2C), suggesting that both types of actin-bundling factor are required to maintain the longitudinal arrangement of shank-localized actin bundles. Strikingly, we found that the angles were decreased in *fim5 plim2a plim2b* pollen tubes when compared with *fim5* pollen tubes (Figure 2C). The disorganized shank-localized actin bundle phenotype was also examined by visualizing transverse sections of pollen tubes (Figure 2D). WT pollen tubes contain both thin and thick actin structures, but these were replaced with intermediate-sized actin structures in *fim5* pollen tubes (Figure 2E). Although the actin structures in *fim5 plim2a plim2b* pollen tubes appear similar to those in *fim5* pollen tubes in terms of area, they are more dispersed (Figure 2E). Surprisingly, the fluorescence intensity of actin structures in *fim5 plim2a plim2b* pollen tubes is significantly higher than that in *fim5* and *plim2a plim2b* pollen tubes (Figures 2D, 2E, and S4). We further found that actin structures are more concentrated at the cortex of *fim5* pollen tube compared with WT, as judged from transverse sections (Figure 2F). The cortex-concentrated actin phenotype is partially suppressed in *fim5 plim2a plim2b* pollen tubes when compared with *fim5* (Figures 2F and S5).These results together suggest that knocking out *PLIM2a* and/or *PLIM2b* alleviates the actin bundle phenotype in the shank region of *fim5* pollen tubes in terms of their fluorescence intensity and spatial distribution (Figure 2G).

**Figure 3:**
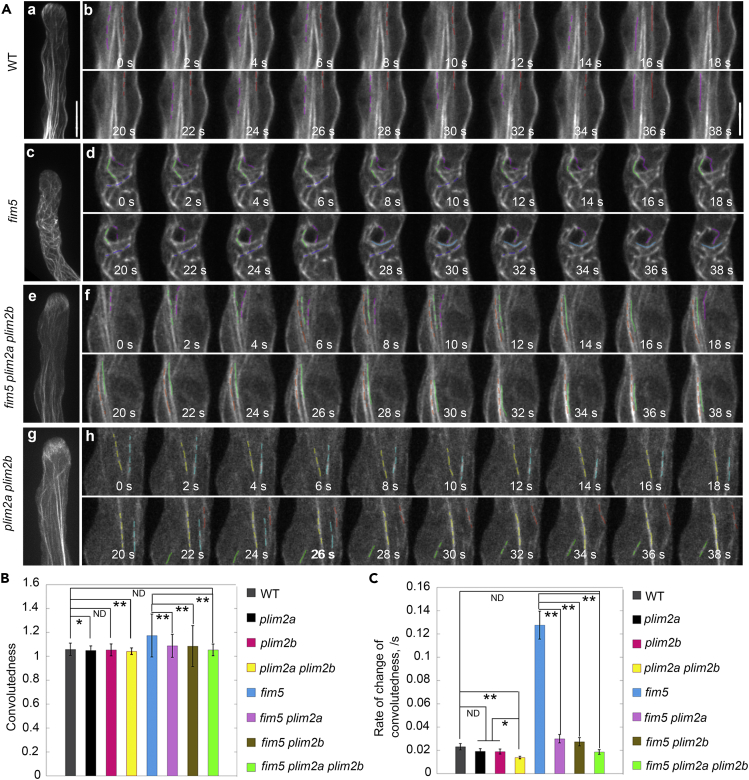
Loss of Function of *PLIM2a* and *PLIM2b* Suppresses the Wavy Actin Bundle Phenotypes within the Shank Region of *fim5* Pollen Tubes (A) Actin filaments decorated with Lifeact-EGFP in pollen tubes. The far left panels are the projection images of the actin filaments in the entire pollen tubes. The right panels are the enlarged time-lapse images of the pollen tubes presented in the far left panels. See also the entire series in Video S1 (WT), Video S2 (*fim5*), Video S3 (*fim5 plim2a plim2b*), and Video S4 (*plim2a plim2b*). Scale bars, 10 μm in the far left panels and 5 μm in the time-lapse images. (B) Quantification of convolutedness in pollen tubes. Values represent mean ± SE, *p < 0.05, **p < 0.01 by Student’s *t*-test. ND, no significant difference. At least three independent experiments were performed. More than 190 actin bundles were measured for each genotype. (C) Quantification of the rate of change of convolutedness in pollen tubes. Values represent mean ± SE, *p < 0.05, **p < 0.01 by Student’s *t*-test. ND, no significant difference. At least three independent experiments were performed. More than 190 actin bundles were measured for each genotype.

To reveal the molecular details underlying the organization of actin structures in the shank, we performed live-cell imaging of actin filament dynamics in the pollen tube. We found that actin bundles arranged longitudinally in the shank region of a WT pollen tube undergo frequent bundling and debundling (Figure 3A [a, b]). By comparison, we found that actin filaments were more curved in the shank of *fim5* pollen tubes and were unable to maintain the longitudinal arrangement (Figure 3A [c, d]), which is consistent with our previous observations (Wu et al., 2010, Zhang et al., 2016a). No obvious disorganization of shank-localized actin bundles was detected in *plim2a*, *plim2b,* and *plim2a plim2b* pollen tubes when compared with WT pollen tubes (Figure 3A [g, h]; Figures S6A and S6B). The actin filaments in the shank of *fim5 plim2a*, *fim5 plim2b,* and *fim5 plim2a plim2b* pollen tubes are straighter than in *fim5* pollen tubes (Figure 3A [e, f]; Figures S6C and S6D). Time-lapse images revealed that actin bundles curved more frequently in *fim5* pollen tubes (Videos S1, S2, S3, and S4), and this was confirmed by measuring the convolutedness and the rate of change of convolutedness of actin filaments as reported previously (Qu et al., 2013, Staiger et al., 2009). Both parameters were reduced significantly in *fim5 plim2a*, *fim5 plim2b,* and *fim5 plim2a plim2b* pollen tubes when compared with *fim5* pollen tubes (Figures 3B and 3C). Thus our data suggest that loss of function of *PLIM2a* and/or *PLIM2b* can suppress the longitudinal actin bundle phenotype in *fim5* pollen tubes.

Video S1. Dynamics of Actin Filaments in the Shank Region of a WT Pollen Tube, Related to Figure 3 Video corresponds to time-lapse images of Lifeact-EGFP-decorated actin filaments in the shank region of a WT pollen tube as shown in Figure 3A. Images were collected at 2-s intervals and are displayed at 5 frames per second (fps).

Video S2. Dynamics of Actin Filaments in the Shank Region of a *fim5* Pollen Tube, Related to Figure 3 Video corresponds to time-lapse images of Lifeact-EGFP-decorated actin filaments in the shank region of a *fim5* pollen tube as shown in Figure 3A. Images were collected at 2-s intervals and are displayed at 5 frames per second (fps).

Video S3. Dynamics of Actin Filaments in the Shank Region of a *fim5 plim2a plim2b* Pollen Tube, Related to Figure 3 Video corresponds to time-lapse images of Lifeact-EGFP-decorated actin filaments in the shank region of a *fim5 plim2a plim2b* pollen tube as shown in Figure 3A. Images were collected at 2-s intervals and are displayed at 5 frames per second (fps).

Video S4. Dynamics of Actin Filaments in the Shank Region of a *plim2a plim2b* Pollen Tube, Related to Figure 3 Video corresponds to time-lapse images of Lifeact-EGFP-decorated actin filaments in the shank region of a *plim2a plim2b* pollen tube as shown in Figure 3A. Images were collected at 2-s intervals and are displayed at 5 frames per second (fps).

### Loss of Function of *PLIM2a* and/or *PLIM2b* Suppresses the Intracellular Trafficking Phenotype in *fim5* Pollen Tubes

**Figure 4:**
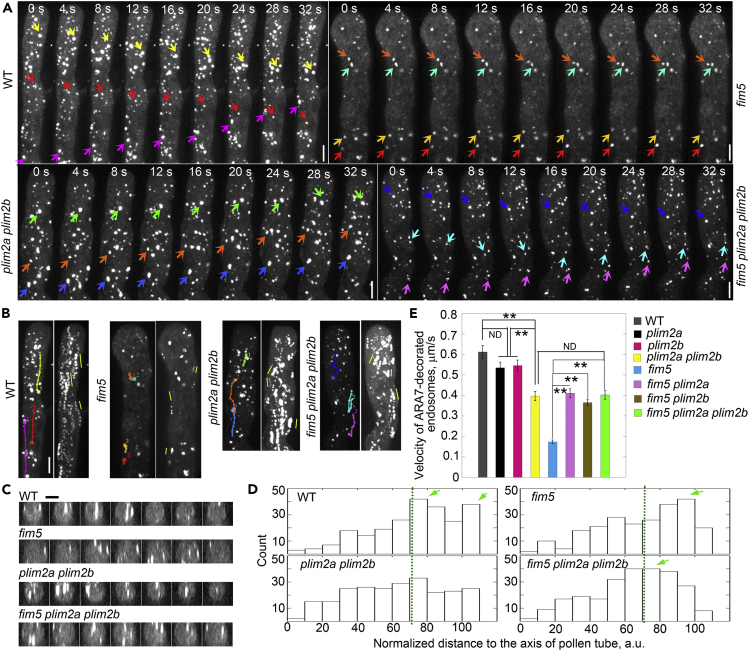
Loss of Function of *PLIM2a* and *PLIM2b* Suppresses the Intracellular Trafficking Phenotype in *fim5* Pollen Tubes (A) Time-lapse micrographs of pollen tubes expressing *Lat52*:*YFP-ARA7*. Different colored arrows indicate the motile YFP-ARA7-decorated endosomes in pollen tubes. Scale bar, 5 μm. (B) Micrographs showing the trajectory of motile YFP-ARA7-decorated endosomes. The colored tracks in the left panel correspond to the endosomes indicated by the same colored arrows shown in (A). In the right panels, five consecutive images in (A) were projected by maximum intensity projection to illustrate the movement of endosomes, as highlighted by yellow lines. See the entire series in Video S5. Scale bar, 5 μm. (C) Transverse sections from the shank region of pollen tubes showing the spatial distribution of YFP-ARA7-decorated endosomes. Scale bar, 5 μm. (D) Histogram of the position of YFP-ARA7-decorated endosomes in transverse sections from the shank region of pollen tubes. The diagram of measuring relative distances of YFP-ARA7-decorated endosomes to the center of the transverse sections in the left panel is similar to that in Figure 2F, except that yellow- and red-colored dots indicate YFP-ARA7-decorated endosomes. The relative distances of YFP-ARA7-decorated endosomes to the center of the transverse sections were plotted. The dashed line indicates the radius at 70.7. Green arrows indicate the relatively prominent peaks in the histograms. At least three independent experiments were performed. More than 240 endosomes were measured for each genotype. (E) Quantification of the velocity of YFP-ARA7-decorated endosomes in pollen tubes. Values represent mean ± SE. **p < 0.01 by Student’s *t*-test. ND, no significant difference. At least three independent experiments were performed. More than 80 endosomes were traced and measured for each genotype.

We next determined the effect of loss of function of *PLIM2a* and/or *PLIM2b* on the intracellular trafficking phenotype in *fim5* pollen tubes. To reveal the defect in the organization of shank-localized longitudinally oriented actin bundles, we selected YFP-ARA7 as the marker to decorate endosomes that move in the shank region but do not invade into pollen tube tips (Zhang et al., 2010). We found that YFP-ARA7-decorated endosomes move rapidly and in a straight line in the shank region of WT pollen tubes (Figures 4A and 4B), whereas they move slowly and irregularly in *fim5* pollen tubes (Figures 4A and 4B), which is consistent with our previous observations (Wu et al., 2010). Interestingly, we found that the pattern of movement of YFP-ARA7-decorated endosomes in *fim5 plim2a plim2b* pollen tubes appears to be similar to that in WT and *plim2a plim2b* pollen tubes but is obviously different from that in *fim5* pollen tubes (Figures 4A and 4B). We also analyzed transverse sections to investigate the spatial distribution of transport paths within the shank region of pollen tubes. We found that the number of YFP-ARA7-decorated endosomes at the cortex increases in *fim5* pollen tubes when compared with WT (Figures 4C and 4D). The abnormal endosome distribution phenotype of *fim5* mutants is alleviated in *fim5 plim2a plim2b* pollen tubes (Figures 4C and 4D). Besides the alteration in transport paths of YFP-ARA7-decorated endosomes in pollen tubes, the reduction in the velocity of movement in *fim5* pollen tubes suggests that the function of actin bundles as molecular tracks is compromised (Figure 4E). Interestingly, the velocity of YFP-ARA7-decorated endosomes was significantly higher in *fim5 plim2a*, *fim5 plim2b,* and *fim5 plim2a plim2b* pollen tubes than in *fim5* pollen tubes (Figure 4E). Thus these data together suggest that loss of function of *PLIM2a* and/or *PLIM2b* alleviates the intracellular trafficking phenotype in *fim5* pollen tubes.

Video S5. Dynamics of ARA7-decorated Endosomes in Pollen Tubes, Related to Figure 4 Video corresponds to time-lapse images of ARA7-decorated endosomes in pollen tubes as shown in Figure 4A. Images were collected at 4-s intervals and are displayed at 3 frames per second (fps).

### PLIM2a/PLIM2b and FIM5 Are Involved in the Regulation of Actin Filament Bundling in Pollen Tubes

To understand how PLIM2a/PLIM2b coordinates with FIM5 in regulating shank-localized longitudinally oriented actin bundles in the pollen tube, we sought to characterize the biochemical functions of PLIM2a/PLIM2b *in vitro* and determine their spatial relationship with FIM5 in the pollen tube. We found that both PLIM2a and PLIM2b were able to generate higher-order actin structures (Figures S7A and S7B) and stabilize actin filaments *in vitro* (Figure S7C), which is consistent with a previous report (Papuga et al., 2010). This result, considered along with other evidence (Han et al., 2013, Thomas et al., 2006, Thomas et al., 2007), suggests that bundling of actin filaments is a general feature of LIM domain-containing proteins in plants. We found that the actin filament bundling frequency was reduced in *fim5*, *plim2a*, *plim2b,* and *plim2a plim2b* pollen tubes when compared with WT (Figure S7D). This suggests that the bundling activity of PLIM2a and PLIM2b is biologically significant and that PLIM2a and PLIM2b act redundantly in this process. In addition, significant reduction in actin filament bundling was detected in *fim5 plim2a plim2b* pollen tubes when compared with either *fim5* or *plim2a plim2b* pollen tubes (Figure S7D). This suggests that two types of actin-filament-bundling factor, FIM5 and PLIM2a/PLIM2b, function redundantly in regulating actin filament bundling. Again, this is consistent with the biochemical evidence that FIM5 and PLIM2s are bona fide actin-bundling factors.

### FIM5 and PLIM2a/PLIM2b Decorate Filamentous Actin Structures in Pollen Tubes

**Figure 5:**
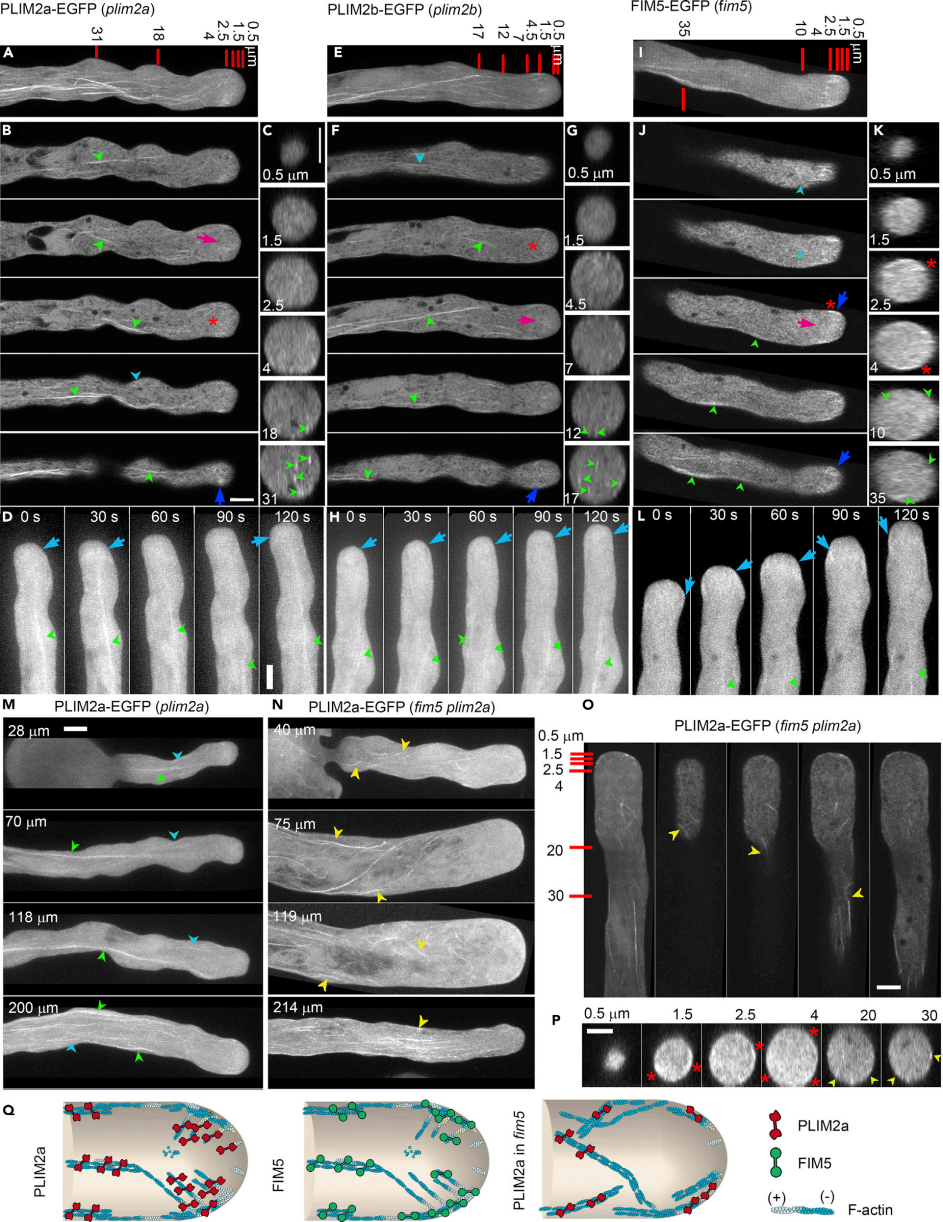
PLIM2a/PLIM2b and FIM5 Decorate Longitudinal Actin Bundles in Pollen Tubes (A–C) PLIM2a-GFP decorates actin filaments throughout the entire pollen tube. The upper panel is the projection of the pollen tube (A). The lower panels show the longitudinal sections (B) and transverse sections (C). (D) Time-lapse images showing the localization of PLIM2a-GFP in the pollen tube. (E–G) PLIM2b-GFP decorates actin filaments throughout the entire pollen tube. The upper panel is the projection of the pollen tube (E). The lower panels show the longitudinal (F) and transverse sections (G). (H) Time-lapse images showing the localization of PLIM2b-GFP in the pollen tube. (I–K) FIM5-GFP decorates actin filaments throughout the entire pollen tube. The upper panel is the projection of the pollen tube (I). The lower panels show the longitudinal (J) and transverse sections (K). (L) Time-lapse images showing the localization of FIM5-GFP in the pollen tube. (M) Micrographs of *plim2a* pollen tubes expressing *PLIM2apro*:*PLIM2a-EGFP*. Pollen tubes of different lengths are presented. Thick and thin actin filamentous structures are indicated by green and blue arrowheads, respectively. (N) Micrographs of *fim5 plim2a* pollen tubes expressing *PLIM2apro*:*PLIM2a-EGFP*. Pollen tubes of different lengths are presented. Disorganized filamentous actin structures are indicated by yellow arrowheads. (O and P) Longitudinal (O) and transverse sections (P) of a *fim5 plim2a* pollen tube expressing *PLIM2apro*:*PLIM2a-EGFP*. The signals locate at the cortex of the apical region in the pollen tube, as indicated by red asterisks. Yellow arrowheads indicate disorganized filamentous structures labeled by PLIM2a in the shank. Arrowheads and arrows indicate shank- and apical-region-localized actin structures, respectively. Green and blue arrowheads indicate thick and thin filamentous actin structures, respectively. Yellow arrowheads indicate disorganized filamentous structures. Dark blue and magenta arrows indicate cortical and internal filamentous actin structures within the growth domain of pollen tubes, respectively. Light blue arrows indicate actin structures in time-lapse images. The bright apical actin structures are indicated by red asterisks. (Q) Schematic showing the distribution of PLIM2a and FIM5 in a WT pollen tube (left and middle panels) and PLIM2a in a *fim5* pollen tube (right panel). Scale bars, 5 μm in all images. Scale bars in (B), (C), and (D) are for (A–L). Scale bar in (M) is for (M) and (N).

To determine the intracellular distribution of PLIM2a and PLIM2b and their spatial relationship with FIM5 in the pollen tube, we generated PLIM2a-EGFP and PLIM2b-EGFP fusion constructs driven by their native promoters and found that they are functional because they can rescue the *plim2a plim2b* mutant phenotype (Figure S8A). To accurately identify their intracellular localization, we examined the distribution of PLIM2a-EGFP and PLIM2b-EGFP signals in the corresponding *plim2a* and *plim2b* single mutants. We found that both PLIM2a-EGFP and PLIM2b-EGFP decorate actin filaments throughout the entire pollen tube (Figures S8B, 5A and 5E), including the prominent longitudinal actin bundles within the shank region (Figures 5A–5H). Consistent with previous reports (Wu et al., 2010, Zhang et al., 2016a), FIM5 decorates shank-localized longitudinal actin bundles besides subapical actin filaments in pollen tubes (Figures 5I–5L). These data suggest that FIM5 and PLIM2a/PLIM2b decorate shank-localized longitudinal actin bundles in the pollen tube. We next selected PLIM2a as the representative protein and examined the effect of *FIM5* loss of function on its localization in pollen tubes of different lengths. In *PLIM2apro*:*PLIM2a-EGFP*;*plim2a* pollen tubes of various lengths, PLIM2a decorates both thick and thin longitudinal actin bundles (Figure 5M). In short *fim5* pollen tubes, PLIM2a decorated disorganized actin bundle structures (Figure 5N), but in long *fim5* pollen tubes, the PLIM2a-decorated actin structures had a more normal longitudinal arrangement in the shank region (Figure 5N). This was also confirmed by visualization of different longitudinal and transverse optical sections of *fim5* pollen tubes expressing *PLIM2apro*:*PLM2a-EGFP* (Figure 5O and 5P). Compared with *pLIM2apro*:*PLM2a-EGFP*;*plim2a* pollen tubes (Figure 5A), the actin structures in transverse optical sections of *PLIM2apro*:*PLM2a-EGFP*;*fim5 plim2a* pollen tubes were dispersed (Figure 5P). These data suggest that FIM5 and PLIM2a/PLIM2b decorate longitudinal actin bundles in the shank of pollen tubes, and the disorganized actin bundles in *fim5* pollen tube are decorated by PLIM2a (Figure 5Q). However, we found that loss of function of *FIM5* does not obviously alter the amount of either PLIM2a-EGFP or PLIM2b-EGFP in pollen (Figure S9), which suggests that the translation of PLIM2a-EGFP and PLIM2b-EGFP does not increase in proportion to their transcription. These data together suggest that PLIM2a/PLIM2b likely contributes to the actin phenotype in *fim5* pollen tubes by replacing FIM5 in binding to actin filaments.

### PLIM2a/PLIM2b and FIM5 Generate Distinct Actin Structures and Compete for Binding to Actin Filaments *In Vitro*

**Figure 6:**
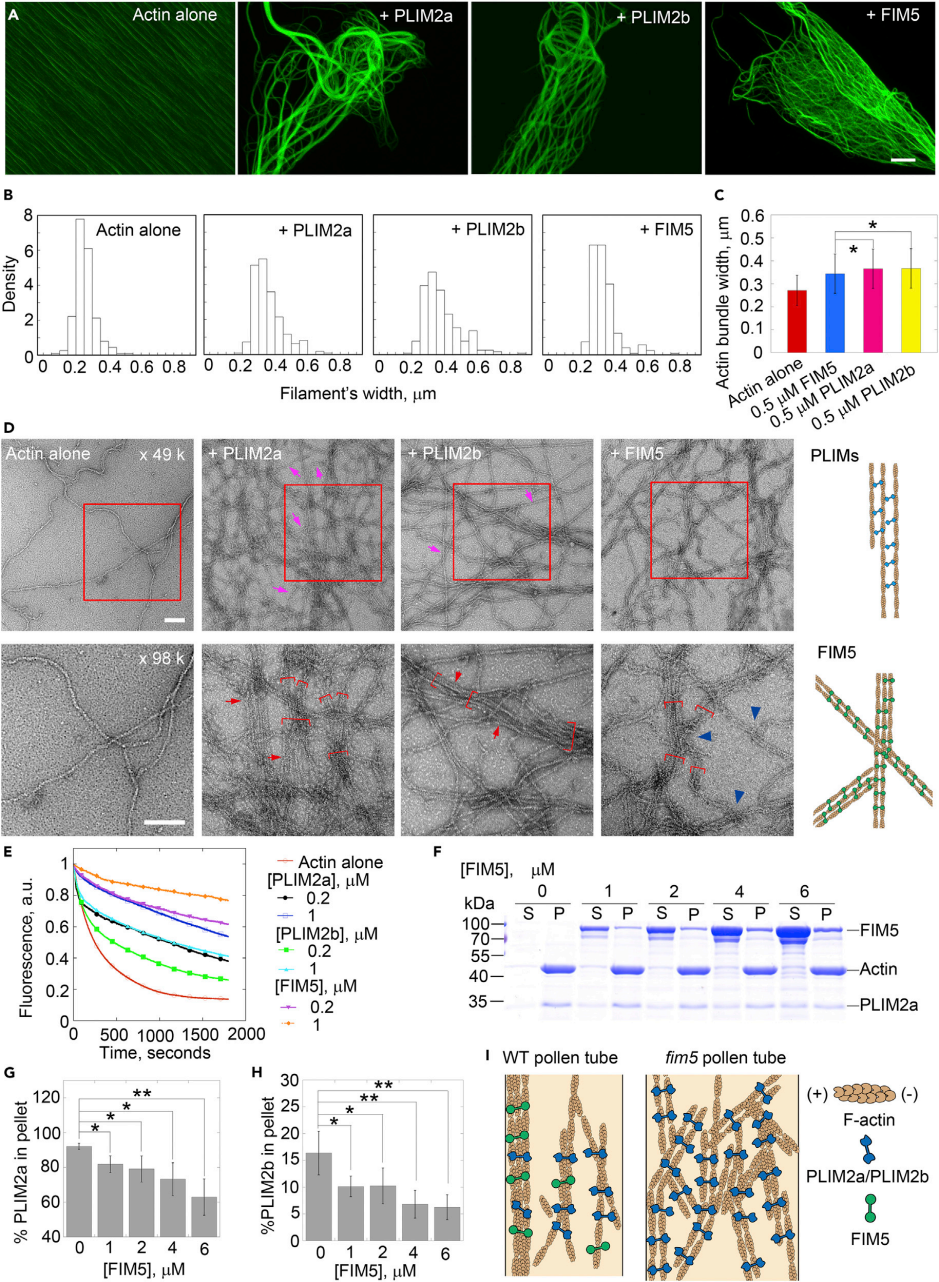
PLIM2s and FIM5 Generate Distinct Higher-Order Actin Structures with Differential Stability, and FIM5 Inhibits the Binding of PLIM2a and PLIM2b to Actin Filaments *In Vitro* (A) Micrographs of rabbit actin filaments in the absence or presence of recombinant *Arabidopsis* FIM5, PLIM2a, or PLIM2b. Actin filaments (4 μM) were incubated with 0.5 μM PLIM2a, 0.5 μM PLIM2b, or 0.5 μM FIM5 and subsequently stained with rhodamine phalloidin. Scale bar, 20 μm. (B) Histogram of the width of actin structures in the absence or presence of 0.5 μM PLIM2a, 0.5 μM PLIM2b, or 0.5 μM FIM5 shown in (A). [Actin], 4 μM. More than 90 actin bundles were measured for each combination. (C) Quantification of the width of actin bundles from (A). More than 90 actin bundles were measured for each combination. *p < 0.05 by Student’s *t*-test. (D) Electron microscopic images of actin filaments in the absence or presence of FIM5, PLIM2a, or PLIM2b. The boxed regions in the top panels are enlarged in the bottom panels. [Actin], 4 μM; [PLIM2a], 0.5 μM; [PLIM2b], 0.5 μM; [FIM5], 0.5 μM. Actin bundles are indicated by red square brackets. Red arrows indicate adjacent actin filaments in one bundle with a longer distance in the presence of PLIM2a or PLIM2b. Purple arrows indicate single actin filaments in the presence of PLM2a or PLIM2b. The cross-linking of thin actin bundles is indicated by blue arrowheads. Bars, 100 nm. The right panel is a schematic model of the actin structures generated by PLIM2a/PLIM2b or FIM5. (E) FIM5 and PLIM2s stabilize actin filaments in a dilution-mediated actin depolymerization assay. Preassembled actin filaments (5 μM, 50% pyrene labeled) were diluted 25-fold in Buffer G, and actin depolymerization was monitored by tracing the changes in pyrene fluorescence. (F) SDS-PAGE image of F-actin cosedimentation experiments in the presence of FIM5 and PLIM2a. [PLIM2a] was kept constant at 2 μM, whereas [FIM5] increased in dosage from 0 to 6 μM. (G) Quantification of the amount of PLIM2a in the pellet in the presence of various concentrations of FIM5 shown in (F). Values represent mean ± SD; *n* = 3; *p < 0.05, **p < 0.01 by Student’s *t*-test. (H) Quantification of the amount of PLIM2b in the pellet in the presence of various concentrations of FIM5. Values represent mean ± SD; *n* = 5; *p < 0.05, **p < 0.01 by Student’s *t*-test. (I) Schematic describing the functional coordination of FIM5 and PLIM2a/PLIM2b in the regulation of actin bundling in a WT pollen tube (left panel) and the consequence of loss of FIM5 function on actin filament organization in a *fim5* pollen tube (right panel). Based on the evidence presented in this article, we propose that PLIM2a/PLIM2b coordinates with FIM5 in binding to and bundling actin filaments to maintain the normal bundling and organization of actin filaments in the shank region of WT pollen tubes. Within *fim5* pollen tubes, the FIM5-binding sites on the actin filaments are occupied by PLIM2a/PLIM2b, leading to the formation of disorganized and thicker actin bundles (right panel).

To determine whether FIM5 and PLIM2a/PLIM2b are biochemically distinct, we performed side-by-side comparisons of the higher-order actin structures generated by FIM5 and PLIM2a/PLIM2b *in vitro*. Direct visualization of actin structures by florescence light microscopy showed that actin structures generated by PLIM2a/PLIM2b were more curved and heavy, whereas those generated by FIM5 were more thin and straight (Figure 6A). This was confirmed by measurements showing that PLIM2a/PLIM2b-generated actin bundles are wider than FIM5-generated actin bundles (Figures 6B and 6C). Direct visualization by electron microscopy (EM) showed that PLIM2a/PLIM2b generates thick and loose actin bundles, whereas FIM5 only generates thin and compact actin bundles (Figure 6D). However, these thin actin bundles can be cross-linked into big actin structures in the presence of FIM5 (Figure 6D). This presumably explains why both heavy and thin actin structures disappear in *fim5* pollen tubes and are replaced with uniform actin bundles of intermediate size. In addition, we found that actin filaments are loosely packed within PLIM2a/PLIM2b-generated actin bundles and individual actin filaments can be detected in the EM images (Figure 6D). In contrast, actin filaments are tightly linked within FIM5-generated actin bundles (Figure 6D). This might explain why FIM5-decorated actin structures are more stable than PLIM2a/PLIM2b-decorated actin structures, as evidenced by dilution-mediated actin depolymerization experiments (Figure 6E). These results suggest that actin structures generated by FIM5 and PLIM2a/PLIM2b have differential biochemical properties *in vitro*.

Next, we wondered whether FIM5 and PLIM2a/PLIM2b share binding sites on actin filaments and whether they will affect each other in binding to actin filaments. Based on the actin phenotype in *fim5* pollen tubes, we hypothesized that FIM5 prevents the binding of PLIM2a/PLIM2b to actin filaments in WT pollen tubes and the FIM5 binding sites on actin filaments are occupied by PLIM2a/PLIM2b in the absence of FIM5. If this is the case, FIM5 should inhibit the binding of PLIM2a/PLIM2b to actin filaments. We therefore performed a high-speed F-actin cosedimentation assay in the presence of various concentrations of FIM5 and a constant concentration of PLIM2a/PLIM2b. We found that FIM5 indeed inhibited the binding of PLIM2a/PLIM2b to actin filaments in a dose-dependent manner (Figures 6F–6H). These data suggest that FIM5 can prevent the binding of PLIM2a/PLIM2b to actin filaments. Interestingly, using PLIM2b as the representative PLIM protein, we found that it can inhibit the binding of FIM5 to actin filaments *in vitro* (Figure S10). These data together suggest that FIM5 and PLIMs likely interfere with each other's binding to actin filaments in pollen tubes.

## Discussion

Here we demonstrate that the balance between FIM5 and PLIM2a/PLIM2b is required to maintain the normal bundling and proper organization of longitudinal actin bundles within the shank region of pollen tubes. Based on *in vitro* and *in vivo* data, we propose a simplified model describing the coordination of FIM5 with PLIM2a/PLIM2b in maintaining longitudinally arranged actin bundles within the shank region of the pollen tube (Figures 6I). Our study suggests that, besides bundling and stabilizing actin filaments (Wu et al., 2010), a key function of FIM5 *in vivo* is to prevent the excessive binding of other bundling factors (e.g., PLIM2a/PLIM2b in this study) to actin filaments. Our results also suggest that maintenance of the balance between two different types of actin-bundling factor with distinct biochemical activity is crucial for the organization and proper function of shank-localized actin bundles in pollen tubes.

### The Balance between Two Biochemically Distinct Actin-Bundling Factors Is Necessary to Regulate Shank-Localized Actin Bundles

Our data suggest that coordination of biochemically distinct actin-bundling factors is biologically relevant. In line with our findings, previous studies suggest that cellular structures containing higher-order actin structures make use of specific sets of actin-bundling factors. For instance, it was shown that the appearance of normal actin bundles and bristles requires the sequential action of at least two different actin-bundling factors, including forked and fascin, during bristle formation in *Drosophila* (Tilney et al., 1998, Wulfkuhle et al., 1998), whereas generation of cytoplasmic actin bundles requires a different combination of actin-bundling factors (quail and fascin) in *Drosophila* nurse cells (Cant et al., 1994, Mahajan-Miklos and Cooley, 1994). Our data thus add another piece of evidence that employment of different combinations of actin-bundling factors with distinct biochemical activities represents a general theme in the regulation of actin bundles in different organisms. *In vitro* observations suggest that PLIM2a/PLIM2b generates thick but loose actin structures and FIM5 generates thin and compact actin bundles that tend to be cross-linked to form heavy structures (Figures 6A–6D). This provides a biochemical explanation for the existence of both thin and thick actin bundle structures in WT pollen tubes (Figure 2A). Given that FIM5 can inhibit the binding of PLIM2a/PLIM2b to actin filaments (Figures 6F–6H), more PLIM2a/PLIM2b will bind to actin filaments in the absence of FIM5 and consequently generate intermediate-sized actin bundles in *fim5* pollen tubes (Figure 2A). Besides the thicker actin bundle phenotype, we noticed that actin bundles bend more frequently and are more curved in *fim5* pollen tubes (Figure 3; Zhang et al., 2016a), which is presumably because PLIM2a/PLIM2b-decorated actin bundles have reduced rigidity. In support of this, we found that PLIM2a/PLIM2b-generated actin bundles are more curved *in vitro* (Figure 6A), which to some extent explains why actin bundles became disorganized in *fim5* pollen tubes (Figures 2 and 3; Wu et al., 2010). Our data suggest that FIM5 and PLIM2a/PLIM2b need to be maintained at a certain ratio *in vivo*. Our study enriches our understanding of the regulation of the generation and maintenance of actin bundle structures within cells.

### Potential Biological Consequence of Differential Decoration of Actin Structures with Different Actin-Bundling Factors

In regulating the organization of the actin cytoskeleton in the pollen tube, FIM5 has two crucial and biologically significant roles: bundling and stabilizing actin filaments and limiting the binding of other bundling factors like PLIM2a/PLIM2b. In the future, *in vitro* reconstitution of the interactions between different actin-bundling factors and actin filaments using biomimetic assays and single-molecule multicolor total internal reflection fluorescence microscopy may provide insights into their coordination. Decoration of actin bundles with different actin-bundling factors might confer distinct biochemical and biophysical properties upon the filaments besides the difference in their morphology. It was shown recently that decoration of actin filaments with different actin-bundling proteins causes differential sorting of other actin-bundling factors, as evidenced by the findings that fimbrin and espin bind to fascin but not to alpha-actinin bundles (Winkelman et al., 2016). In addition, coordination of different actin-bundling factors may confer certain biochemical and biophysical properties upon actin filaments that may fine-tune the activities of some important players within the actin turnover machinery (e.g., ADF and its cofactors; Allwood et al., 2002, Augustine et al., 2011, Augustine et al., 2008, Chen et al., 2002, Jiang et al., 2017, Shi et al., 2013, Zheng et al., 2013) to regulate actin dynamics in pollen tubes. It was shown that competition between FIM1 and tropomyosin differentially antagonizes the action of cofilin (Skau and Kovar, 2010). Our preliminary results, which show that FIM5-and PLIM2a/PLIM2b-decorated actin structures have differential resistance to dilution-mediated actin depolymerization (Figure 6E), partially support the hypothesis that different actin-bundling factors modulate the properties of actin filaments. Further *in vitro* reconstitution of actin filaments decorated with either FIM5 or PLIM2a/PLIM2b in the presence of factors that promote actin turnover may provide more insights into this aspect of actin biology.

Given that one of the major functions of longitudinal actin bundles is providing molecular tracks for myosin motors, decoration of actin bundles with different actin-bundling factors may affect the motor activity of myosins. It was shown that the tropomyosin isoform Tpm2p, but not Tpm1p, inhibits myosin-based retrograde movement of actin cables in yeast (Huckaba et al., 2006). This points to the possibility that matching of actin-bundling factors with myosin motors is crucial for myosin-based motility. We found that intracellular trafficking was downregulated to different extents within pollen tubes derived from loss-of-function mutants of *fim5* and *plim2a*/*plim2b* (Figure 4), which suggests that decoration of actin bundles with different actin-bundling factors at a certain ratio may indeed fine-tune the activity of myosin-based movement. *In vitro* reconstitution of myosin-based motility in the presence of different actin-bundling factors at certain ratios might provide clues to this. In addition, besides functioning as molecular motor, myosins were also reported to be directly involved in regulating the organization of actin filaments by pulling them. Indeed, it was shown that Class XI myosins are directly involved in the regulation of actin organization in plant cells (Cai et al., 2014, Peremyslov et al., 2010). A parallel report showed that myosin activity is crucial for the condensation of actin cables within the actin ring in yeast (Laporte et al., 2012). In future, it needs to be considered whether the match of actin-bundling factors with myosin motors is important for the role of myosins in regulating the organization of actin bundles in the pollen tube. It will also be worth examining whether the differential coordination of FIM5 and PLIM2a/PLIM2b with different myosin motors is implicated in regulating the organization of longitudinal actin bundles in pollen tubes.

In summary, we demonstrate that the balance between FIM5 and PLIM2a/PLIM2b is crucial for the regulation of the construction and organization of shank-localized actin bundles in pollen tubes. Our study thus enriches our understanding of the molecular mechanism underlying the generation of molecular railways that support various intracellular transportation events in pollen tubes.

### Limitation of the Study

In this study, we demonstrate that the balance between actin-bundling factors is crucial for the proper spatial distribution and organization of actin structures in pollen tubes. However, how pollen tubes fine-tune the balance between actin-bundling factors during pollen tube growth and what is the precise biological significance of maintaining the balance between actin-bundling factors in pollen tubes remain to be elucidated.

## Methods

All methods can be found in the accompanying Transparent Methods supplemental file.

## Supplemental Information

Document S1. Transparent Methods, Figures S1–S10, and Table S1
