# Supplementary material for: The Balance between Actin-Bundling Factors Controls Actin Architecture in Pollen Tubes
Source: iScience. 2019 May 25;16:162–76. doi: 10.1016/j.isci.2019.05.026 (PMC6556835; doi:10.1016/j.isci.2019.05.026)
Supplement: Document S1. Transparent Methods, Figures S1–S10, and Table S1 [file mmc1.pdf]

ISCI, Volume *16*

Supplemental Information

The Balance between Actin-Bundling Factors Controls Actin Architecture in Pollen Tubes

Ruihui Zhang, Xiaolu Qu, Meng Zhang, Yuxiang Jiang, Anbang Dai, Wanying Zhao, Dai Cao, Yaxian Lan, Rong Yu, Hongwei Wang, and Shanjin Huang

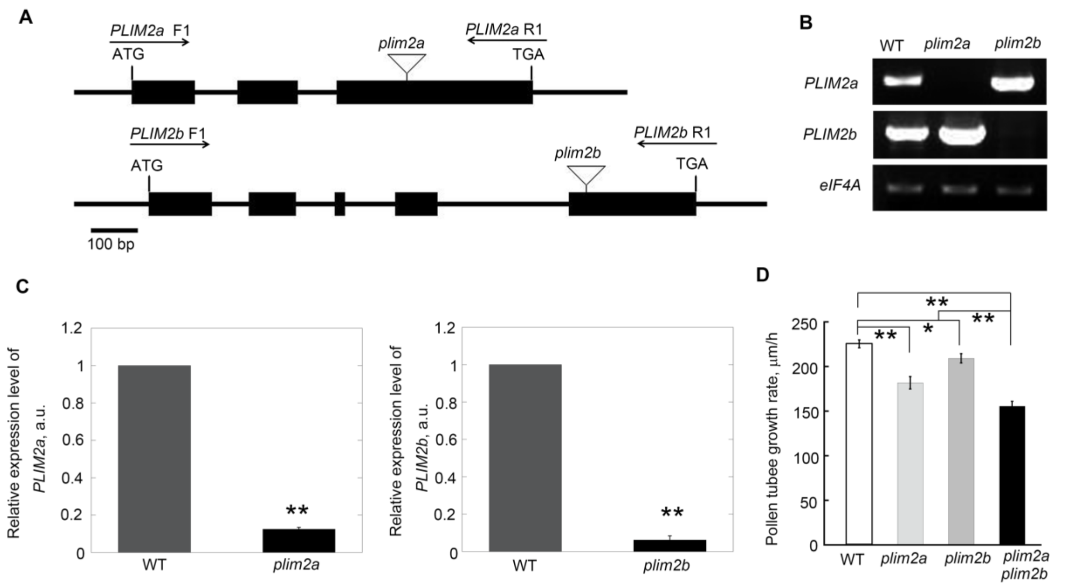

Figure S1. *PLIM2a* and *PLIM2b* Redundantly Regulate Pollen Tube Growth, Related to Figure 1

(**A**) The physical structure of the *PLIM2a* and *PLIM2b* genes. The black lines and boxes indicate the introns and exons, respectively. Salk_122321, *plim2a*; GABI_537H11, *plim2b*. F1 and R1 indicate the positions of primers used for RT-PCR analysis. The inverted triangles indicate the position of the T-DNA insertion sites in the T-DNA mutants.

(**B**) Determination of the relative levels of *PLIM2a* or *PLIM2b* transcripts in *plim2a* and *plim2b* mutants by semi-quantitative (q) RT-PCR. *eIF4A* was used as the internal loading control.

(**C**) Determination of the relative levels of *PLIM2a* and *PLIM2b* transcripts in the corresponding mutants by qRT-PCR. $^{**}P < 0.01$ by Student's *t*-test.

(**D**) Quantification of pollen tube growth rates. More than 60 pollen tubes were measured for each genotype. At least 3 independent experiments were performed and one typical result was shown. Values represent mean ± SE; $^{*}P < 0.05$, $^{**}P < 0.01$ by Student's *t*-test. ND, no significant difference.

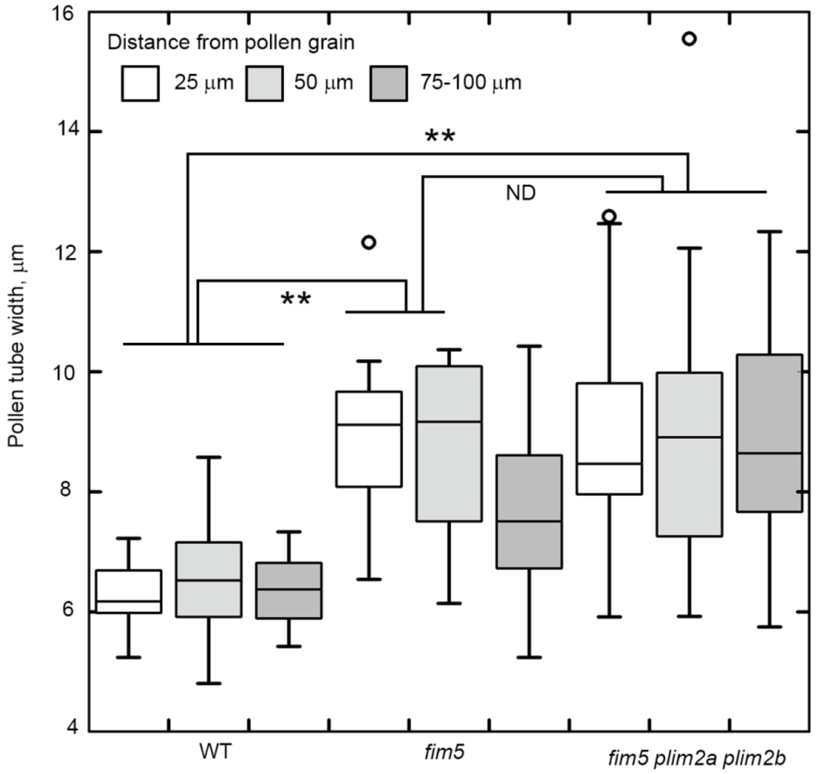

Figure S2. Loss of function of *PLIM2a* and *PLIM2b* does not alter pollen tube width in *fim5*, Related to Figure 1

The widths of pollen tubes of different lengths were measured and plotted. More than 13 pollen tubes were measured for each genotype. The statistical comparisons were performed with the Kruskal-Wallis test using SPSS13 software; ** P <0.01.

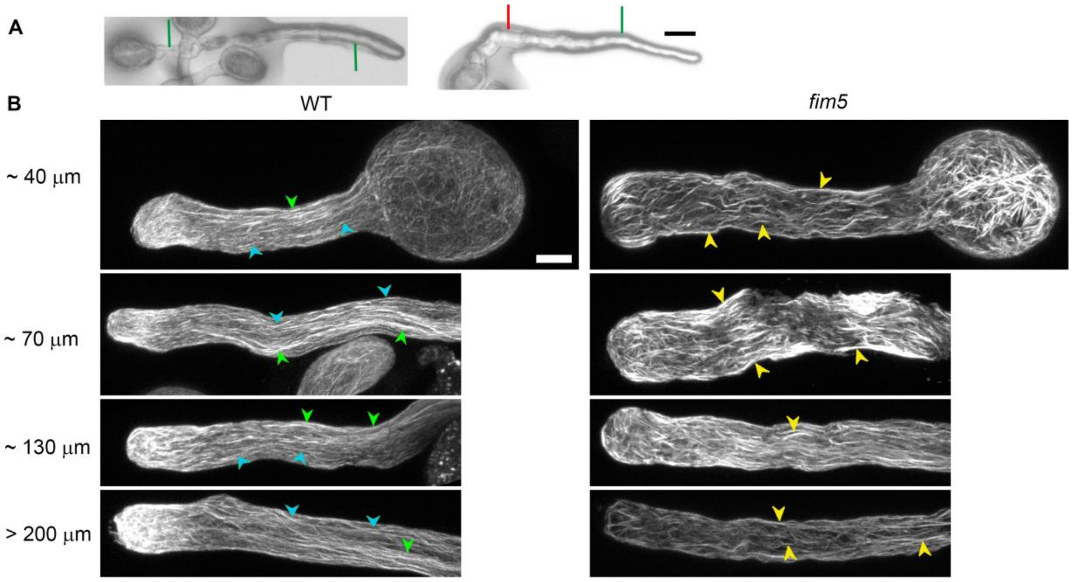

Figure S3. Visualization of Actin Filaments within WT and *fim5* Pollen Tubes of Different Lengths, Related to Figure 2

(**A**) Micrographs of WT (left) and *fim5* (right) pollen tubes. Green lines indicate regions of normal width in WT pollen tube and in the *fim5* pollen tube at the late growth phase. The red line indicates the swollen region in the early growth phase of a *fim5* pollen tube. Bar = 20 µm.

(**B**) Actin filaments in WT and *fim5* pollen tubes of different lengths. Actin filaments were revealed by staining with Alexa-488-phalloidin in fixed pollen tubes of different lengths. Heavy actin bundles and fine actin structures are indicated by green and blue arrowheads, respectively, in WT pollen tubes. Uniform intermediate-sized but disorganized actin bundles in *fim5* pollen tubes are indicated by yellow arrowheads. Bar = 5 µm.

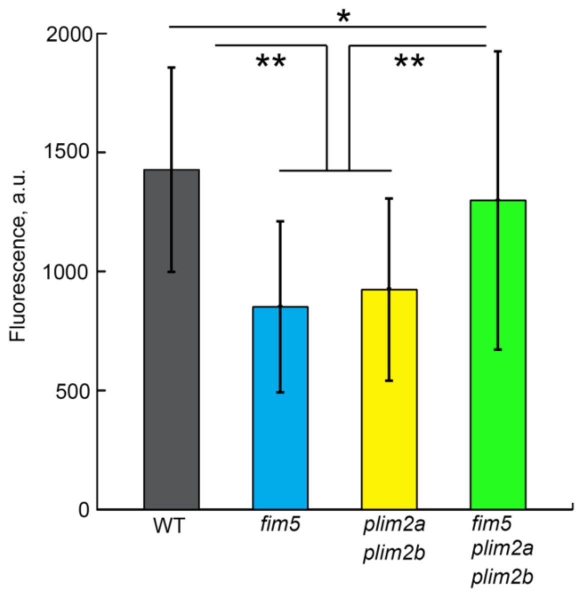

Figure S4. Quantification of Fluorescence Intensity of Actin Structures within Transverse Sections derived from the shank region of pollen tubes, Related to Figure 2

Average fluorescent intensity of actin filaments within the transverse sections of pollen tubes were measured and plotted. Values represent mean ± SD. * P < 0.05, ** P < 0.01 by Student's t-test, *n* > 179. At least 3 independent experiments were performed and one typical result was shown.

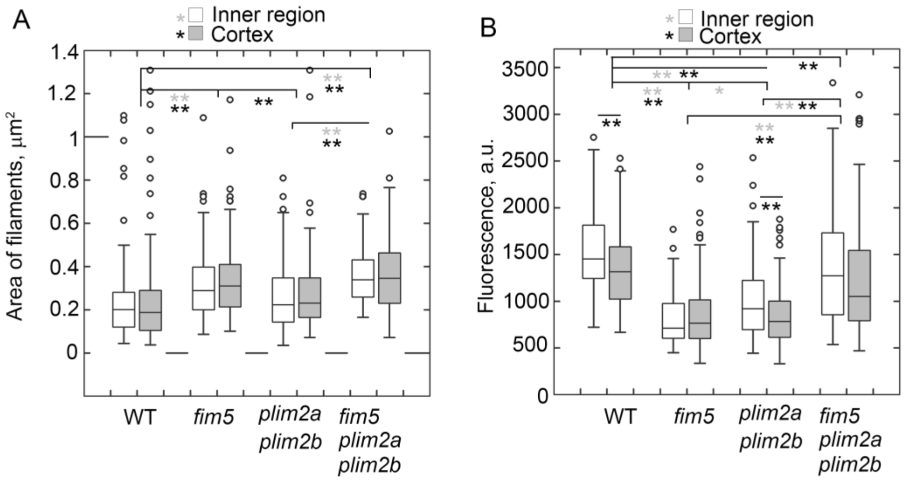

Figure S5. Quantification of Area and Intensity of Actin filaments at the Cortex and Inner Region within Transverse Sections of Pollen Tubes, Related to Figure 2

As indicated in the legend of Figure 2F, every transverse section was divided into two parts, the outer annulus and inner circle, which were defined as the cortex and inner region, respectively. The average area (**A**) and average fluorescence intensity (**B**) of actin filaments within the outer annulus and inner circle of transverse sections were measured and plotted. The statistical analysis was conducted with the Kruskal-Wallis test using SPSS13 software; *P < 0.05, **P < 0.01. *n* > 170. At least 3 independent experiments were performed and one typical result was shown.

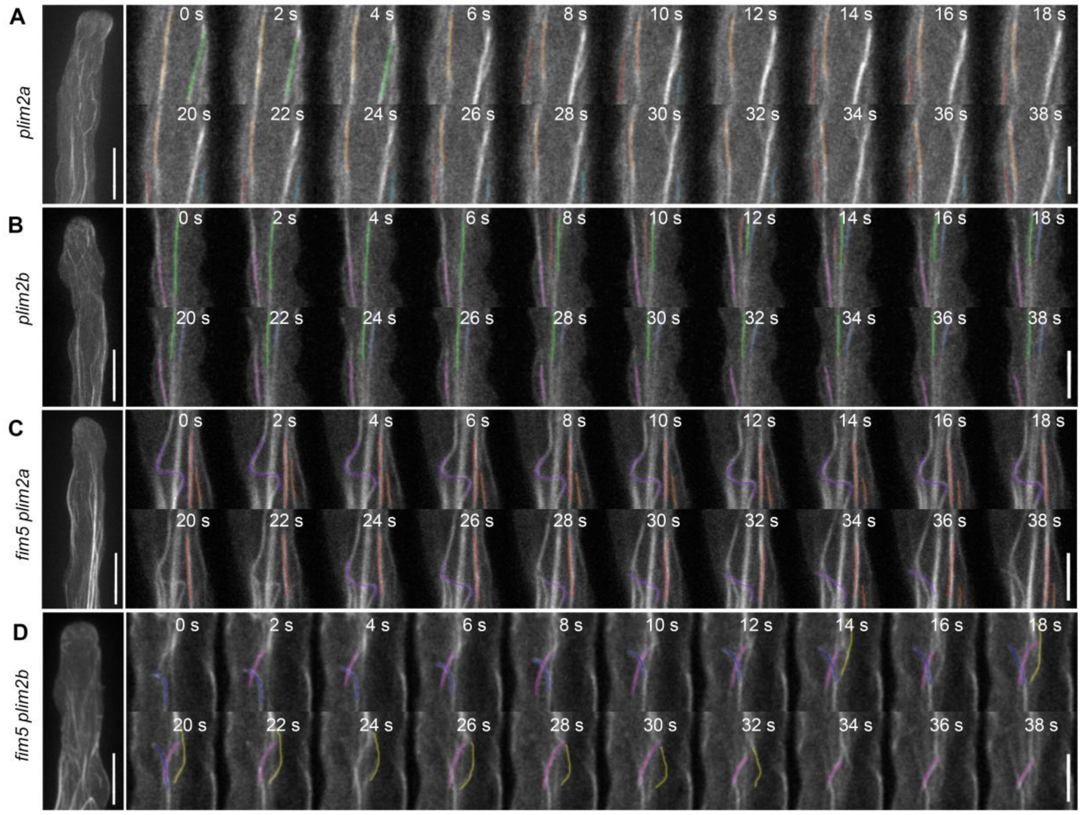

Figure S6. Loss of Function of *PLIM2a* or *PLIM2b* Suppresses the Wavy Actin Filaments Phenotype in *fim5* Pollen Tubes, Related to Figure 3

Actin filament dynamics in *plim2a* (**A**), *plim2b* (**B**), *fim5 plim2a* (**C**) and *fim5 plim2b* (**D**) pollen tubes. Actin filaments were decorated with Lifeact-EGFP in pollen tubes. The left panels are the projection images of entire pollen tubes from the indicated mutants. The right panels are time-lapse images. Bars in the left panels and right panels are 10 μm and 5 μm, respectively.

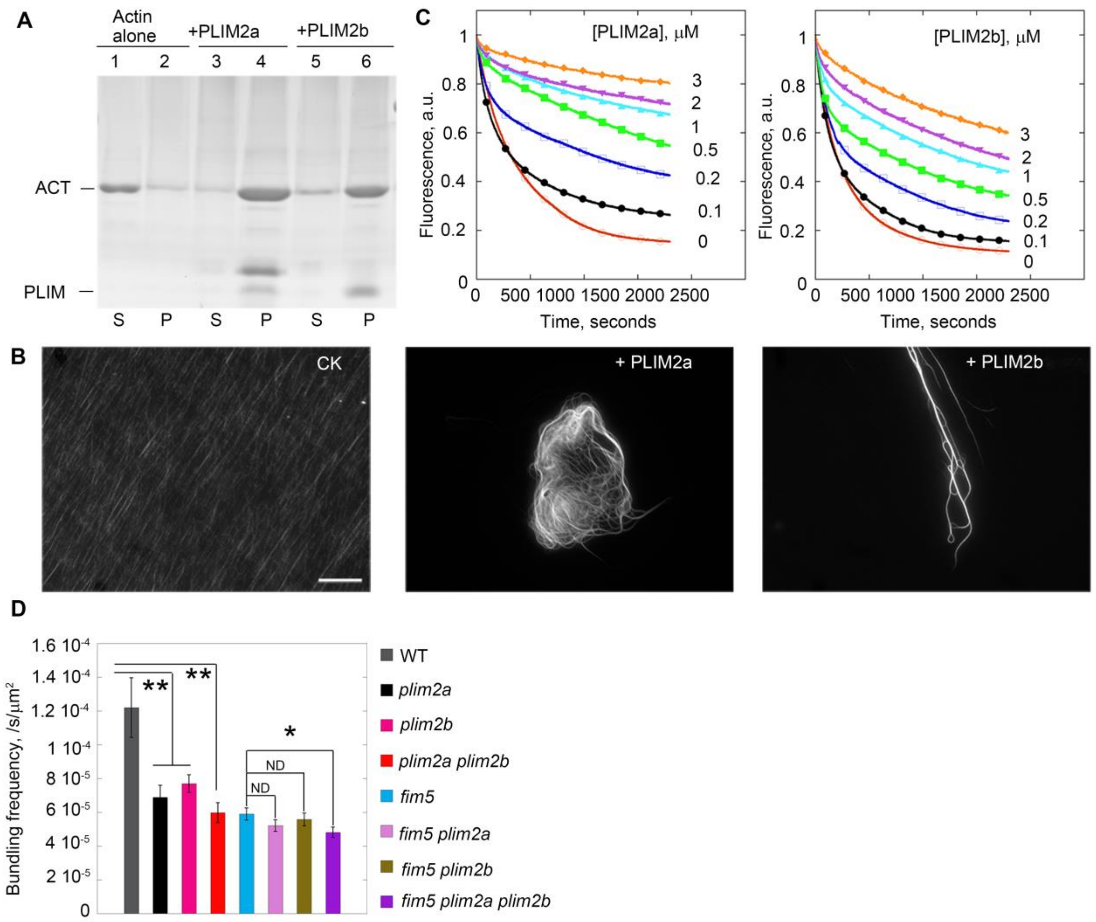

Figure S7. PLIM2a and PLIM2b are *bona fide* Actin Bundlers That Stabilize Actin Filaments *in vitro*, and Their Loss of Function Affects Actin Bundling in Pollen Tubes, Related to Figures 5 and 6

(**A**) Low speed F-actin cosedimentation assay showing that PLIM2a and PLIM2b bundle actin filaments.

(**B**) Micrographs of actin filament structures. Actin filaments (4 μM) in the absence or presence of 1 μM PLIM2a or PLIM2b were stained with rhodamine-phalloidin. Bar = 20 μm.

(**C**) PLIM2a and PLIM2b stabilize actin filaments in a dose-dependent manner *in vitro*. Preassembled actin filaments (5 μM, 50% pyrene-labeled) were diluted 25 fold in the presence of various concentrations of PLIM2a (left panel) and PLIM2b (right panel). Actin depolymerization was monitored by tracing the changes in pyrene fluorescence.

(**D**) Quantification of actin filament bundling frequency in pollen tubes. *P < 0.05, **P < 0.01 by Student's *t*-test. ND, no significant difference. *n* > 16. At least 3 independent experiments were performed.

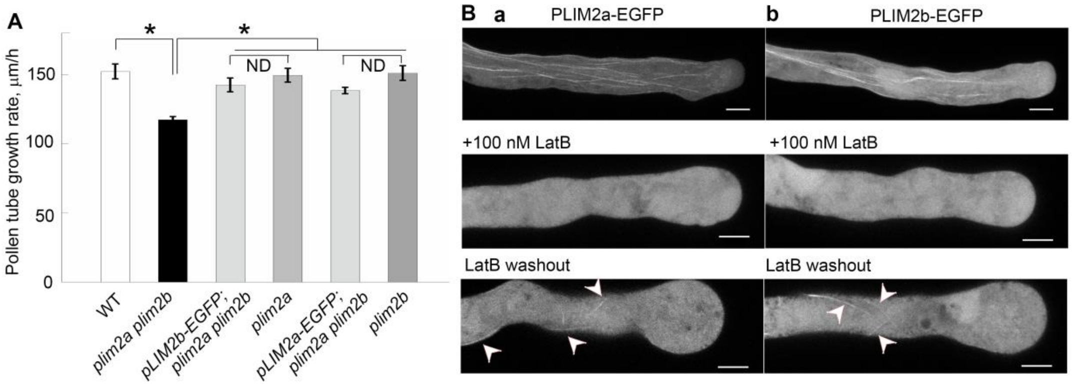

Figure S8. PLIM2a-EGFP and PLIM2b-EGFP Decorate Actin Filaments in Pollen Tubes, Related to Figure 5

(**A**) PLIM2a-EGFP or PLIM2b-EGFP rescues the reduced pollen tube growth rate in *plim2a plim2b*. PLIM2a-EGFP and PLIM2b-EGFP represent the transgenic plants *PLIM2apro:PLIM2a-EGFP;plim2a plim2b* and *PLIM2bpro:PLIM2b-EGFP;plim2a plim2b*, respectively. *P < 0.05 by Student's *t*-test. ND, no significant difference. *n* > 26. At least 3 independent experiments were performed.

(**B**) PLIM2a- and PLIM2b-decorated filamentous structures are actin filaments. Pollen tubes derived from the transgenic plants *PLIM2apro:PLIM2a-EGFP;plim2a* and *PLIM2bpro:PLIM2b-EGFP;plim2b* were subjected to treatment with the actin depolymerizing agent LatB (100 nM). Filamentous structures were broken down upon LatB treatment and reformed after washout of LatB. White arrowheads indicate the recovered filamentous structures. Bar = 5 μm.

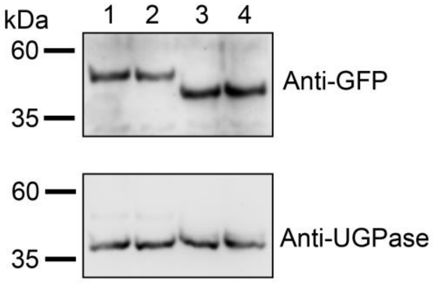

Figure S9. Determination of the Relative Amount of PLIM2a and PLIM2b in Pollen by Western-blot Analysis, Related to Figure 5

Total proteins were extracted from *Arabidopsis* pollen as described in the Method section. Lane 1, PLIM2a-EGFP (*plim2a*); lane 2, PLIM2a-EGFP (*plim2a fim5*); lane 3, PLIM2b-EGFP (*plim2b*) and lane 4, PLIM2b-EGFP (*plim2b fim5*). Information describing the transgenic plants can be found in Figure 5. Upper panel, western blot probed with anti-GFP antibody; lower panel, western blot probed with anti-UGPase antibody. This experiment was repeated three times and the typical result was shown.

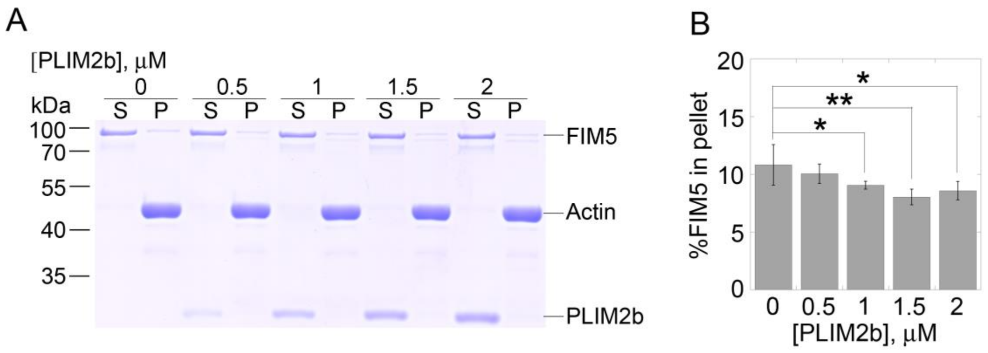

Figure S10. PLIM2b Inhibits the Binding of FIM5 to Actin Filaments *in vitro*, Related to Figure 6

(**A**) SDS-PAGE image of F-actin cosedimentation experiments in the presence of FIM5 and PLIM2a. [FIM5] was kept constant at 0.5 μM whereas [PLIM2b] increased in dosage from 0-2 μM.

(**B**) Quantification of the amount of FIM5 in the pellet in the presence of various concentrations of PLIM2b shown in (**A**). Values represent mean ± SD; $n = 3$, $^{*}P < 0.05$ and $^{**}P < 0.01$ by Student's *t*-test.

Table S1. Primers used in this study, Related to Figures 1, 2, 3, 4, 5 and 6

primer name	primer sequence
plim2a LP	CGTGTCCTAAGACAAGAACCG
plim2a RP	CAACAGATTCTCTCCAAAAAGAGC
Salk LB1.3	CGTGTCCTAAGACAAGAACCG
plim2b LP	AAAGGAGTGGCTCTCGTCTTC
plim2b RP	TGGAACACAAGACAAGTGTGC
GABI LB	ATATTGACCATCATACTCATTGC
fim5 LP	TTTAGGACGGTGAGGCATATG
fim5 RP	GCGAGTGTGATCTCAAGTTCC
wisCp745	AACGTCCGCAATGTGTTATTAAGTTGTC
PLIM2a F1	AAAGACCGAGAAGCCCAA
PLIM2a R1	AGAGTGAGTCAAAGGACAACCA
PLIM2b F1	GACTCACTCCTCCTACGCCT
PLIM2b R1	TCTAGACTCTGAAACGCCA
q*VLN2* F	GAAGCCCGGGATTACTCTCA
q*VLN2* R	CTTCAGCATGGGTGTCTAGGA
q*VLN5* F	TCGGTAAAGATTCCAGCCA
q*VLN5* R	GAACCCTGAAGCAACTCCAC
q*FIM4* F	TCTGCCAAGGCTATTGGGTG
q*FIM4* R	CACCAGCTGGGGAGTTTTCT
q*PLIM2c* F	TGACAAGACGGTATACGTTATG
q*PLIM2c* R	TTGGAAATTCTTGCTGAAATT
q*ADF5* F	TTACCGGTGGATGATTGTC
q*ADF5* R	AAGTTCATAGTGAATCCCT
q*CROLIN* F	CAGAGCTTCTGCAAGTAAG
q*CROLIN* R	GCTGTTTCATCCACGTT
eIF4A F	CGATGTGCAGCAAGTCTCTC
eIF4A R	CTCCCGAACCTTCCACTTCT
gPLIM2b F	AAGCTTTGCAAAACCAATGGATGAAAATGTGT
gPLIM2b R	GAGCTCAGACTCTGAAACGCCATTCTCT
gPLIM2a F	AAGCTTAATTAGGATCCGGACCAGGA
gPLIM2a R	CAGGAGCTCAGACTCAACGACCGGCTC
PLIM2a F	CGCGAATTCATGAGCTTTACCGGTACAC
PLIM2a R	CGAAGCTTGCTTTCAACAACCG
PLIM2b F	CGCGAATTCATGAGCTTTACAGGCACC
PLIM2b R	CGAAGCTTGCTCTCGCTCACGC

Underlined sequences represent restriction enzyme recognition sites

Supplemental Sequences

>PLIM2a

ATGAGCTTTACCGGTACACTGGACAAGTGCAAAGCCTGCGACAAGACCGTTTATGTGATGGACCTGCTGAC
CCTGGAAGGCAACACCTACCACAAGAGTTGCTTCCGTTGCACCCACTGCAAAGGCACACTGGTGATCAGCA
ACTATTCTAGCATGGATGGCGTGCTGTATTGCAAGCCGCACTTCGAGCAGCTGTTCAAAGAAAGCGGCAATT
ACAGCAAGAACTTCCAGGCCGGCAAGACCGAAAAACCGAACGATCATCTGACCCGTACCCCGAGCAAACT
GAGCAGCTTTTTCAGCGGTACCCAGGACAAATGCGCCACCTGTAAGAAAACCGTGTACCCGCTGGAGAAG
GTGACCATGGAAGGCGAGAGCTACCACAAAACCTGTTTTCGCTGCACCCATAGCGGCTGCCCGCTGACCCA
TAGCAGCTATGCAAGCCTGAACGGCGTGCTGTACTGTAAGGTGCACTTCAACCAGCTGTTCCTGGAGAAAG
GCAGCTATAACCACGTGCATCAGGCAGCCGCAAATCATCGCCGTAGCGCAAGCAGCGGTGGTGCCAGTCCT
CCGAGCGATGATCATAAACCGGATGACACCGCCAGCATTCCGGAAGCCAAAGAAGACGATGCCGCACCGG
AAGCAGCAGGCGAAGAAGAACCGGAACCGGTTGTTGAAAGCTAA

>PLIM2b

ATGAGCTTTACAGGCACCCTGGATAAGTGCAACGTGTGCGACAAGACCGTTTATGTGGTGGACATGCTGAG
CATCGAGGGCATGCCGTACCACAAAAGTTGCTTCCGCTGTACCCACTGTAAGGGCACCCTGCAGATGAGCA
ACTATAGCAGCATGGACGGCGTGCTGTATTGCAAGACCCATTTTGAACAGCTGTTTAAAGAAAGCGGCAATT
TTAGCAAGAACTTCCAGCCGGGCAAAACCGAAAAACCTGAACTGACCCGCACCCCGAGCAAAATTAGCAG
CATCTTCTGCGGCACCCAAGACAAATGCGCCGCATGCGAAAAGACCGTGTACCCGCTGGAAAAGATCCAGA
TGGAGGGCGAATGCTTCCACAAAACCTGCTTTCGCTGTGCCCACGGTGGTTGTACCCTGACCCACAGCAGC
TACGCCAGCCTGGACAGCGTGTTATATTGCCGCCACCACTTCAACCAGCTGTTCATGGAGAAGGGCAACTAT
GCACACGTTCTGCAGGCCGCCAATCATCGCCGTACCGCAAGTGGTAATACCCTGCCTCCGGAACCGACCGA
AGATGTGGCCGTGGAGGCCAAAGAAGAAAATGGCGTGAGCGAGAGCTAA

Transparent Methods

Plant Materials and Growth Conditions

T-DNA insertion lines Salk_122321 and GABI_537H11 were designated as *plim2a*, and *plim2b*, respectively. The genotyping of *plim2a* and *plim2b* was performed with primer combinations *PLIM2a* LP/*PLIM2a* RP/Salk LB 1.3 and *PLIM2b* LP/*PLIM2b* RP/GABI LB (Table S1), respectively. Information about *fim5-1*, which was used as *fim5* in this study, has been presented previously (Wu et al., 2010). The *Arabidopsis* Columbia-0 (Col-0) ecotype was used as wild type (WT), and plants were grown in the growth chamber under a 16-h-light/8-h-dark photoperiod at 22 °C.

qRT-PCR Analysis

Total pollen RNA was extracted by TRIzol reagent (Invitrogen), and 3 μg RNA was subsequently used for reverse transcription by MMLV reverse transcriptase (Promega) to synthesize cDNA. Transcripts of *PLIM2a*, *PLIM2b*, *VLN2*, *VLN5* and *FIM4* were amplified with primer pairs *PLIM2a* F1/*PLIM2a* R1, *PLIM2b* F1/*PLIM2b* R1, q*VLN2* F/q*VLN2* R, q*VLN5* F/q*VLN5* R and q*FIM4* F/q*FIM4* R (Table S1), respectively, using an Applied Biosystems® 7500 fast Real-Time PCR System. *eIF4A* was amplified with *eIF4A* F/*eIF4A* R (Table S1) as the internal loading control. The transcript levels of target genes were calculated by the $2^{\Delta Ct}$ method (Livak and Schmittgen, 2001), in which $\Delta Ct = Ct_{(eIF4A)} - Ct_{(gene)}$.

Pollen Germination and Pollen Tube Growth Measurements

Arabidopsis pollen germination and pollen tube growth measurements were carried out as previously described (Chang and Huang, 2015). In detail, fresh pollen was collected from newly opened *Arabidopsis* flowers and spread onto the surface of solid pollen germination medium (GM: 1 mM $Ca(NO_3)_2$, 1 mM $CaCl_2$, 1 mM $MgSO_4$, 0.01% (w/v) H_3BO_3, 18% sucrose (w/v), pH 7.0, solidified with 0.8% (w/v) agarose) and cultured at 28 °C. Pollen germination was quantified after culturing for 3 hrs. For the measurement of pollen tube growth rate, images of pollen tubes were captured after culturing for 2 h and 2.8 h. The lengths of the same pollen tube were measured at

those two time points, and the length difference between those two time points was divided by the time interval to yield the average pollen tube growth rate. Since *fim5* pollen tubes grow slowly, images of pollen tubes were acquired at 4.5 h and 5.3 h after germination. The experiment was repeated at least three times.

Complementation and Visualization of Intracellular Localization of PLIM2a and PLIM2b in Pollen Tubes

To generate *PLIM2apro:PLIM2a-EGFP* and *PLIM2bpro:PLIM2b-EGFP* constructs, the genomic sequences of PLIM2a and PLIM2b were amplified with primer pairs *gPLIM2a* F/*gPLIM2a* R and *gPLIM2b* F/*gPLIM2b* R (Table S1), respectively. They were subsequently moved into pCambia1301-*EGFP* restricted with *BamH*I/*Sac*I to generate pCambia1301-*gPLIM2a-EGFP* and pCambia1301-*gPLIM2b-EGFP*, respectively. The plasmids pCambia1301-*gPLIM2a-EGFP* and pCambia1301-*gPLIM2b-EGFP* were transformed into *plim2a plim2b* plants using the floral dip method (Clough and Bent, 1998) to generate the transgenic plants pCambia1301-*gPLIM2a-EGFP;plim2a plim2b* and pCambia1301-*gPLIM2b-EGFP;plim2a plim2b*, respectively. To observe the intracellular localization of PLIM2a and PLIM2b, the transgenic plants pCambia1301-*gPLIM2a-EGFP;plim2a plim2b* and pCambia1301-*gPLIM2b-EGFP;plim2a plim2b* were initially crossed with *plim2a* or *plim2b* to finally obtain pCambia1301-*gPLIM2a-EGFP;plim2a* and pCambia1301-*gPLIM2b-EGFP;plim2b* transgenic plants. Pollen tubes derived from pCambia1301-*gPLIM2a-EGFP;plim2a* and pCambia1301-*gPLIM2b-EGFP;plim2b* plants were observed under an Olympus FV1000 laser scanning confocal microscope equipped with a ×100 objective. Pollen tubes of different lengths were selected for microscopic observation and image acquisition. The samples were excited with a 488 nm laser and emission wavelength was set in a 500 nm–600 nm range. To demonstrate that the filamentous structures decorated with PLIM2a-EGFP or PLIM2b-EGFP are actin filaments, pollen tubes were subjected to treatment with 100 nM latrunculin B (LatB). For subsequent washout experiments, the tubes were washed with liquid

pollen germination medium to remove the LatB.

Total Protein Extraction from *Arabidopsis* Pollen and Western-blot Analysis

The extraction of total protein from *Arabidopsis* pollen was performed as described previously (Chang and Huang, 2015, 2017). Specifically, pollen derived from *plim2a*, *plim2a fim5*, *plim2b* and *plim2b fim5 Arabidopsis* plants was collected and ground in liquid nitrogen. Protein extraction buffer (100 mM HEPES, pH 7.5, 5 mM EGTA, 10 mM DTT, 5% glycerol, 0.1% NP-40, 1 mM PMSF) was added to the ground pollen, mixed and centrifuged at 16,000 g for 30 min at 4 °C. The supernatant of *Arabidopsis* total protein was collected and separated on 10% SDS-polyacrylamide gels for western blotting. The western-blot analysis was performed according to the manufacturer's instructions (Bio-Rad). The anti-GFP polyclonal antibody (CW0087, CWBio) and HRP-conjugated goat anti-rabbit IgG antibody (CW0103, CWBio) were used at 1:3000 and 1:10000 dilution, respectively. The signal was detected by Thermo Scientific SuperSignal West Dura Extended Duration Substrate (34075, Thermo Fisher Scientific). After imaging, the PVDF membrane was stripped with stripping buffer (1.5% glycine, 0.1% SDS, 1% Tween20, pH 2.2), and subsequently reprobed with anti-UGPase antibody (AS05 086, Agrisera) at 1:4000 dilution as the loading control.

Visualization of F-actin Organization in Fixed Pollen Tubes and Actin Filament Dynamics in Living Pollen Tubes

The organization of actin filaments in fixed *Arabidopsis* pollen tubes was revealed by staining with Alexa-488 phalloidin as described previously (Zhang et al., 2010a). In detail, pollen tubes were fixed with 300 mM N-(maleimidobenzoyloxy)-succinimide in liquid pollen GM for 1 h and washed with TBS-T (50 mM Tris, 200 mM NaCl, 400 mM Sucrose and 0.05% Nonidet P-40, pH 7.4) for three times. Subsequently, the pollen was stained with 200 nM Alexa-488 phalloidin (Molecular Probes) overnight at 4 °C. To quantify the organization of actin filaments within the shank region of the pollen tube, the angles formed between actin filaments and the pollen tube growth

axis were measured in the longitudinal sections. The organization of actin structures was also analyzed by measuring their cross-sectional area and fluorescence intensity in transverse sections of pollen tubes, as well as their distances to the centers of the transverse sections. Considering that the pollen tube diameter varies between different pollen tubes, the distances were divided by the radius to obtain the normalized distance values. To trace the dynamics of the actin cytoskeleton in living pollen tubes, actin filaments were decorated with Lifeact-EGFP as described previously (Qu et al., 2013; Vidali et al., 2009). Image acquisition with an Olympus BX51 microscope equipped with an Andor Revolution XDh spinning disk confocal system was performed as described previously (Chang and Huang, 2015). The bundling and debundling frequencies of actin filaments were quantified according to previously published methods (Zheng et al., 2013).

Visualization and Quantification of YFP-ARA7-decorated Vesicles in Pollen Tubes

YFP-ARA7 was used as a marker to decorate endosomes in the pollen tube as described previously (Zhang et al., 2010b). It was introduced into *plim2a*, *plim2b* and *plim2a plim2b* by crossing them with transgenic *Arabidopsis* plants harboring *Lat52:YFP-ARA7*. T3 homozygous plants after self-segregation were used for the subsequent analysis. Time-lapse images were collected by spinning disk confocal microscopy as described above. To trace the movement of ARA7-decorated endosomes, the time-lapse images were processed by ImageJ software with an ImageJ plugin ‘MtrackJ’ as described previously (Meijering et al., 2012). Over 80 particles from more than 10 pollen tubes were traced and measured.

Protein Production

To improve the expression level of recombinant PLIM2a and PLIM2b in *E. coli*, the codons of *Arabidopsis* PLIM2a and PLIM2b were optimized before the coding sequences were moved into the prokaryotic expression vector. The sequences of PLIM2a and PLIM2b after codon optimization are shown in Supplemental Sequences.

Using the optimized coding sequences as templates, the PCR products were amplified with primer pairs *PLIM2a* F/*PLIM2a* R and *PLIM2b* F/*PLIM2b* R (Table S1). They were subsequently moved into pET23a to generate the pET23a-*PLIM2a* and pET23a-*PLIM2b* plasmids, and the plasmids were transformed into the *E. coli* Tuner (DE3) pLysS strain. The expression of PLIM2a or PLIM2b was induced by the addition of 0.4 mM isopropyl β-D-thiogalactopyranoside overnight at 16 °C. The recombinant PLIM2a and PLIM2b were purified with Nickel-sepharose according to the manufacturer's instructions. The protein was dialyzed against 10 mM Tris, pH 8.0, then aliquoted, flash frozen in liquid N_2 and stored in a freezer at -80 °C. Recombinant *Arabidopsis* FIM5 protein was generated as described previously (Wu et al., 2010). Actin was isolated from acetone powder of rabbit skeletal muscle (Spudich and Watt, 1971) and monomeric Ca-ATP-actin was further purified using chromatography on Sephacryl S-300 at 4 °C in Buffer G (5 mM Tris-HCl, pH 8.0, 0.2 mM ATP, 0.1 mM $CaCl_2$, 0.5 mM DTT, 0.1 mM NaN_3) (Pollard, 1984). Actin was labeled on Cys-374 with pyrene iodoacetamide in order to monitor the kinetic process of actin polymerization and depolymerization (Pollard, 1984).

High-speed F-actin Cosedimentation Assay

The high-speed F-actin cosedimentation assay was performed according to previously published methods (Huang et al., 2005; Kovar et al., 2000). Specifically, to determine whether FIM5 can prevent the binding of PLIM2a or PLIM2b to actin filaments, preassembled muscle actin filaments at 3 μM were initially incubated with 2 μM recombinant *Arabidopsis* PLIM2a or 2 μM PLIM2b for 5 min. Subsequently, various concentrations of FIM5 were added into the reaction mixtures and incubated for 25 min. To determine whether PLIM2b can prevent the binding of FIM5 to actin filaments, the concentration of FIM5 was kept at 0.5 μM and various concentrations of PLIM2b were subsequently added. The reaction mixtures were subsequently centrifuged at 200000g at 4 °C for 30 min, and the supernatant and pellet fractions were separated by SDS-PAGE. The amount of PLIM2a, PLIM2b or FIM5 in the pellet was quantified by densitometry using ImageJ software (http://rsbweb.nih.gov/ij/;

version 1.51).

Confocal Microscopy of Actin Filaments *in vitro*

Actin filaments labeled with rhodamine-phalloidin were observed under the laser scanning confocal microscope. The procedure was essentially the same as previously published methods of visualizing actin filaments by a fluorescence light microscope (Huang et al., 2003). In detail, 4 μM muscle actin in the presence or absence of 0.5 μM recombinant *Arabidopsis* PLIM2a, PLIM2b or FIM5 was assembled in the presence of 1X KMEI (50 mM KCl, 2 mM $MgCl_2$, 1 mM EGTA, 0.2 mM ATP, 0.2 mM $CaCl_2$, 0.5 mM DTT, 3 mM NaN_3, and 10 mM imidazole, pH 7) at room temperature for 30 min. Actin filaments were revealed by staining with equimolar rhodamine-phalloidin (Sigma Aldrich) during polymerization. Actin filaments were visualized under an Olympus FV1000 confocal microscope equipped with a ×100 oil objective (1.42 numerical aperture). The samples were excited with a 488 nm laser and the emission was set in a range of 500-600 nm for the acquisition of images.

Visualization of Actin Filaments with Electronic Microscopy

Negatively stained actin filaments and actin bundles generated by PLIM2a, PLIM2b or FIM5 were visualized by electron microscopy. In detail, 2 μM pre-polymerized F-actin was gently mixed with 0.5 μM PLIM2a, 0.5 μM PLIM2b or 0.5 μM FIM5, and incubated for 5 min on holey carbon-coated EM copper grids, which were subsequently negatively stained in 2% (w/v) uranyl acetate solution following the procedure described in a previously published method (Liu and Wang, 2011). All the specimens were observed under an FEI Tecnai-T12 electron microscope operated at 120 kV acceleration voltage at corresponding magnification with a range of defocus from 2.0 to 3.0 μm. The electron micrographs were captured by a Gatan Ultrascan4000 4k X 4k CCD camera.

Dilution-mediated Actin Depolymerization Assay

To determine the stability of actin filaments decorated with PLIM2a, PLIM2b or

FIM5, a dilution-mediated actin depolymerization was employed as described previously (Bao et al., 2012). In detail, preassembled muscle actin filaments at 5 μM (50% pyrene-labeled) were incubated with various concentrations of recombinant *Arabidopsis* PLIM2a, PLIM2b or FIM5 for 5 min at room temperature, and the mixtures were subsequently diluted 25-fold in Buffer G. Actin depolymerization was monitored by tracing the changes in pyrene fluorescence using a QuantaMaster Luminescence QM 3 PH fluorometer (Photo Technology International, Inc.) with the excitation and emission wavelength set at 365 nm and 407 nm, respectively.

Accession Numbers

FIM4 (AT5G55400), *FIM5* (AT5G35700), *PLIM2a* (AT2G45800), *PLIM2b* (AT1G01780), *PLIM2c* (AT3G61230), *VLN2* (AT2G41740), *VLN5* (AT5G57320), *ADF5* (AT2G16700), *CROLIN1* (AT3G28630).

Supplementary references

Bao, C., Wang, J., Zhang, R., Zhang, B., Zhang, H., Zhou, Y., and Huang, S. (2012). Arabidopsis VILLIN2 and VILLIN3 act redundantly in sclerenchyma development via bundling of actin filaments. Plant J *71*, 962-975.

Chang, M., and Huang, S. (2015). Arabidopsis ACT11 modifies actin turnover to promote pollen germination and maintain the normal rate of tube growth. Plant J *83*, 515-527.

Chang, M., and Huang, S. (2017). Rapid Isolation of Total Protein from Arabidopsis Pollen. Bio Protoc *7*, e2227.

Clough, S.J., and Bent, A.F. (1998). Floral dip: a simplified method for Agrobacterium-mediated transformation of Arabidopsis thaliana. Plant J *16*, 735-743.

Huang, S., Blanchoin, L., Kovar, D.R., and Staiger, C.J. (2003). Arabidopsis capping protein (AtCP) is a heterodimer that regulates assembly at the barbed ends of actin filaments. J Biol Chem *278*, 44832-44842.

Huang, S., Robinson, R.C., Gao, L.Y., Matsumoto, T., Brunet, A., Blanchoin, L., and Staiger, C.J. (2005). Arabidopsis VILLIN1 generates actin filament cables that are resistant to depolymerization. Plant Cell *17*, 486-501.

Kovar, D.R., Staiger, C.J., Weaver, E.A., and McCurdy, D.W. (2000). AtFim1 is an actin filament crosslinking protein from Arabidopsis thaliana. Plant J *24*, 625-636.

Liu, X., and Wang, H.W. (2011). Single particle electron microscopy reconstruction of the exosome complex using the random conical tilt method. J Vis Exp.

Livak, K.J., and Schmittgen, T.D. (2001). Analysis of relative gene expression data using real-time quantitative PCR and the 2(T)(-Delta Delta C) method. Methods *25*, 402-408.

Meijering, E., Dzyubachyk, O., and Smal, I. (2012). Methods for cell and particle tracking.

Methods Enzymol *504*, 183-200.

Pollard, T.D. (1984). Polymerization of ADP-actin. J Cell Biol *99*, 769-777.

Qu, X., Zhang, H., Xie, Y., Wang, J., Chen, N., and Huang, S. (2013). Arabidopsis villins promote actin turnover at pollen tube tips and facilitate the construction of actin collars. Plant Cell *25*, 1803-1817.

Spudich, J.A., and Watt, S. (1971). The regulation of rabbit skeletal muscle contraction. J Biol Chem *246*, 4866-4871.

Vidali, L., Rounds, C.M., Hepler, P.K., and Bezanilla, M. (2009). Lifeact-mEGFP reveals a dynamic apical F-actin network in tip growing plant cells. PLoS One *4*, e5744.

Wu, Y., Yan, J., Zhang, R., Qu, X., Ren, S., Chen, N., and Huang, S. (2010). Arabidopsis FIMBRIN5, an actin bundling factor, is required for pollen germination and pollen tube growth. Plant Cell *22*, 3745-3763.

Zhang, H., Qu, X., Bao, C., Khurana, P., Wang, Q., Xie, Y., Zheng, Y., Chen, N., Blanchoin, L., Staiger, C.J*., et al.* (2010a). Arabidopsis VILLIN5, an actin filament bundling and severing protein, is necessary for normal pollen tube growth. Plant Cell *22*, 2749-2767.

Zhang, Y., He, J., Lee, D., and McCormick, S. (2010b). Interdependence of endomembrane trafficking and actin dynamics during polarized growth of Arabidopsis pollen tubes. Plant Physiol *152*, 2200-2210.

Zheng, Y., Xie, Y., Jiang, Y., Qu, X., and Huang, S. (2013). Arabidopsis actin-depolymerizing factor7 severs actin filaments and regulates actin cable turnover to promote normal pollen tube growth. Plant Cell *25*, 3405-3423.
